# Classifying Hawaiian plant species along a habitat generalist-specialist continuum: Implications for species conservation under climate change

**DOI:** 10.1371/journal.pone.0228573

**Published:** 2020-02-07

**Authors:** Alison Ainsworth, Donald R. Drake

**Affiliations:** 1 School of Life Sciences, University of Hawaiʻi at Mānoa, Honolulu, Hawaii, United States of America; 2 Pacific Island Network Inventory and Monitoring Program, National Park Service, Volcano, Hawaii, United States of America; CNRS - Universite de Pau et des Pays de l'Adour - E2S UPPA, FRANCE

## Abstract

Plant communities on tropical high islands, such as the Hawaiian Islands, are predicted to experience rapid climate change, resulting in novel climates. If increased temperature and/or drought exceed plant species’ current tolerances, species that are unable to adapt or shift ranges risk extinction. By definition, habitat generalists have a wide niche breadth and thrive in a variety of habitats, whereas habitat specialists have a narrow niche breadth, and typically thrive under more specific climatic characteristics (e.g., cold). The objectives of this study were to: (1) classify plant species in the Hawaiian Islands along a habitat generalist-specialist continuum; (2) independently test the validity of species rankings, using environmental and biogeographic ranges; and (3) identify species’ life-history traits that predict species location along the continuum. We quantified specialization for 170 plant species using species co-occurrence data from over one thousand plots to rank species’ realized habitat niche breadth using the Jaccard index. The distribution of species along this continuum differed by species biogeographic origin, with endemic plant species ranked on the specialist end and non-native plant species ranked on the generalist end. Habitat specialization rankings also differed for four of nine tested variables (while controlling for biogeographic origin): number of habitat moisture types, minimum elevation, number of Hawaiian Islands, and life form. Life form was the only trait tested that differed across the continuum, with woody species ranked as stronger generalists than herbaceous species; this pattern was particularly evident for non-native species. This indirect method of estimating species’ potential climatic flexibility uses increasingly available large plant community data sets with output rankings which represent species’ realized habitat niches. Identifying species and plant communities that are on the habitat specialist end of the continuum allows for their prioritization in conservation planning, as globally the loss of specialists is an indication of degradation.

## Introduction

Plant communities on tropical high islands, such as the Hawaiian Islands, are expected to experience rapid climate change, resulting in novel climates [1,2]. Not all plant species or communities are equally vulnerable to changing climates [3,4,5] as seen in past individualistic responses by some plant species (cf. [6]) to late Quaternary climate change [7]. If increased temperature and/or drought exceed plant species’ current tolerances, species that are unable to adapt or shift ranges quickly enough risk extinction. Climatically-driven adaptation for modern plants is predicted to be less likely than is seen in the fossil record, because the current rate of climate change is more rapid and populations have limited connectivity owing to anthropogenic land use [8]. Therefore, determining which plant species can persist with rapid climate change is critical for conservation planning.

A species’ ecological niche is the multi-dimensional resource space required to ensure a viable population in a given environment [9]. The fundamental niche includes the full range of environmental conditions and resources in which a species can persist, whereas the realized niche incorporates the influence of other biotic mechanisms, usually reducing niche size (e.g., via competition), but sometimes expanding it (e.g., via facilitation) [9]. Despite its long-recognized importance in ecological theory, niche breadth has proven to be challenging to measure. The definition of niche has varied among authors through time, resulting in conflicting interpretations [10]. Occasionally, fundamental climatic niche breadth has been measured experimentally in controlled settings by identifying species’ physiological limits, but such data are rare, expensive, and typically lack biotic influences [11]. For comparisons among species, niche breadth measures must be quantitative and translatable [12] but they are often described qualitatively, or if quantitative, limited to partial niche space due to small spatial scales [13].

Traditionally, realized niche breadth was measured as abundance or occupancy along environmental gradients [14,15]. These measurements are challenging and can be misleading because it is often difficult to identify which abiotic factors are important, how to control others experimentally, and, if and to what extent, interactions among factors may be important [16]. Plant occurrence data coupled with environmental variables are increasingly used to define climate-niche-based species distribution models (SDMs), but the ability of these models to make reliable/accurate predictions varies depending on the selection of and possible interactions between predictor variables [17,18]. Despite the abundance of recent studies utilizing SDMs, most studies use these models to test predictions about species distribution without actually defining what does and does not constitute suitable habitat for the species [19], or relative niche breadth among species. Alternatively, realized niche breadth can be defined based on habitat delineations (i.e., the number of different habitats a species is found in); however, distinguishing unique habitats is dependent on scale and sample size and habitat boundaries are rarely distinct [15]. Recently, quantitative, continuous measures of habitat specialization were developed that do not require assumptions about abiotic gradients or habitat characteristics; rather, this methodology allows species co-occurrence to define realized niche breadth using beta diversity models [16,20].

Theoretically, a species’ habitat niche breadth should be correlated with environmental characteristics and the species’ biogeographical origin (Table 1). Plant species ranked as habitat generalists should be found in many different habitat moisture types (e.g., dry, mesic, wet) and/or climate zones (e.g., temperatures) or elevations. Plant species ranked as generalists should be more abundant in milder, more productive climates where biotic (e.g., competition) filters tend to be stronger determinants than climate in driving species assembly [21]. Alternatively, in harsh environments (i.e., very dry or wet habitats and/or very hot or cold habitats), climate and facilitation may be strong direct or indirect determinants of distribution and community assembly [22,23,24]. Because elevation is correlated with temperature, similar expectations exist for a correlation between species elevation range and habitat generalists, where species ranked as strong generalists should occur across a wide elevation range. Along elevational gradients, species ranked as habitat specialists are expected to occur in limited elevation ranges. For example, low (low maximum elevation) or high (high minimum elevation), because harsher temperature conditions at these elevations require specialized ecophysiological adaptations [e.g., cold temperature plants with low stature, small leaves, pubescence, slow growth rates [25]] which presumably make these stress-tolerant species less competitive in benign environments [26,27].

**Table 1:** Hypotheses to test the validity of species habitat specialization rankings with known species’ environmental and biogeographic ranges and to identify species’ traits that can be used to predict species location along the habitat specialization continuum.

Type	Variable	Generalist Correlation	Predictions for species ranked as generalists:
Environment	No. of habitat moisture types (1–3)	+	will be found in more habitat moisture types, than those ranked as specialists
Environment	Moisture type*	mesic >wet >dry	will be found in habitats that do not require highly specialized physiological adaptations to survive
Environment	Elevation range	+	will be found across a greater range of elevations than specialists
Environment	Elevation minimum	-	will not be restricted to high elevations where specialized physiological adaptations are necessary to survive cool climates
Environment	Elevation maximum	+	will not be restricted to low elevations where specialized physiological adaptations are necessary to survive warm climates
Biogeographic	Species biogeographic origin*	non-native >indigenous >endemic	are more likely to have recently colonized new habitats (non-natives) or repeatedly colonized multiple habitats (indigenous) than species unique to a defined geographic location, such as an island (endemic)
Biogeographic	No. of Hawaiian Islands (1–6)*	+	are more likely to occur on multiple Hawaiian Islands because they can persist in multiple habitats, including diverse substrates
Life history	Leaf size variability	+	are more likely to have greater leaf size variability
Life history	Life form*	woody >herbaceous	are more likely to be woody than herbaceous life forms
Life history	Dispersal syndrome*	null model	will not differ from those ranked as specialists in seed dispersal syndrome (e.g., animal, wind, ballistic, gravity, no special adaption)

*Categorical variables

Habitat generalists should be more likely to invade new ecosystems because of their greater flexibility to persist in diverse habitats [28,11]. Similarly, species ranked as habitat generalists should be more likely to be found on numerous volcanically derived islands with similar climatic characteristics if these islands represent different primary successional sere or substrate types [i.e., chronosequence of Hawaiian Islands age gradients [29]], assuming dispersal is not limiting. Even habitat fragments may contain more generalists than specialists because generalists are better able to track anthropogenic land-use changes [30].

If consistent correlations between habitat specialization and simple, measurable, plant life-history traits can be quantified, then additional species beyond sampled data sets (e.g., rare species) can be assigned habitat specialization values based solely on these life-history traits [31]. Increasingly, studies have documented tight correlations between specific traits [e.g., plant height, specific leaf area (SLA), seed size] and whole plant performance [32]. We hypothesize that generalist species are more likely to have greater leaf size variability, which increases suitability to varied habitat types. Generalists are more likely to be tall, woody species, than short, herbaceous species because height facilitates light capture. Generalist species are unlikely to differ from specialist species in seed dispersal syndrome (e.g., animal, wind, ballistic, gravity, no special adaption) because all syndromes have some potential for long-distance dispersal and seed or propagule quantity typically offsets potential efficiency of dispersal [33,34].

The Hawaiian Islands offer an advantageous system to test methods for quantifying habitat specialization because of their extreme geographic isolation, steep climatic gradients with wide ranges of vegetation types across limited land area [35], multiple islands with shared species, and relatively simplified disharmonic and impoverished flora [36,37]. Identifying specialized plant species with potentially high sensitivity to future climate change is critical, considering the extreme endemism in this biodiversity hotspot, increasing threats of human-facilitated invasions, and documented warming and drying above global averages [38]. Climate change has been and continues to be actively studied in Hawaiʻi with clear evidence from the past four decades of rapidly rising surface temperatures [39], reduced or altered precipitation patterns [40,41], and expected further warming and drying at high elevations, associated with increased frequency and/or lowering of the trade wind inversion layer [42].

The specific objectives of this study were to: (1) classify relatively common plant species in the Hawaiian Islands along a habitat generalist-specialist continuum using co-occurrence data from vegetation plots; (2) test the validity of species habitat specialization rankings with independent species environmental and biogeographic ranges; and (3) identify species’ life-history traits that can be used to predict species location along the habitat generalist-specialist continuum. Results from these three objectives can be used to predict species vulnerability (or lack of vulnerability) to climate change and thereby guide conservation.

## Materials and methods

### Study area

**Fig 1:**
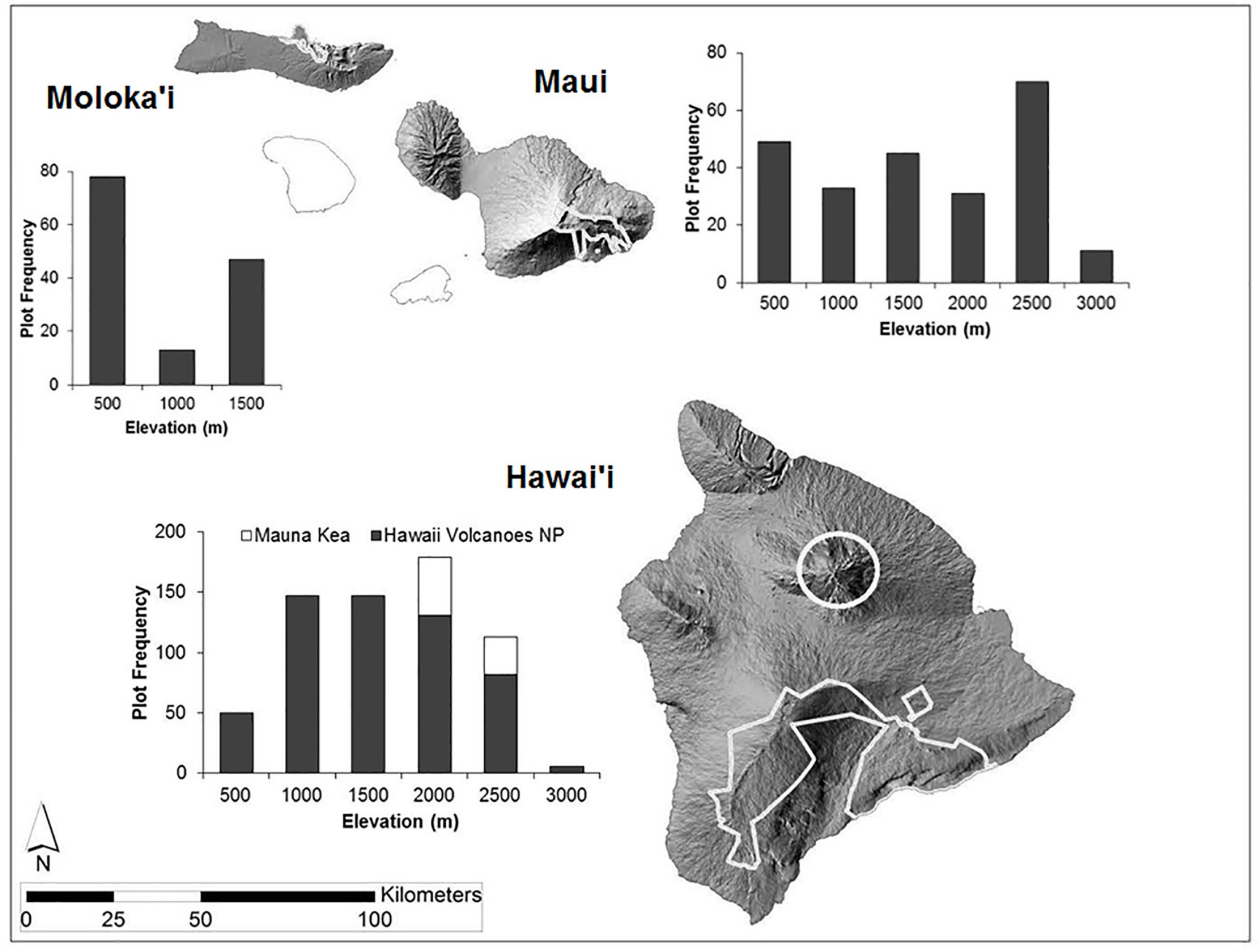
Hawaiian Islands on which plant species co-occurrence samples (1017 plots) were collected between 2004 and 2012. Plots range from sea level to 3000 meters within Hawaiʻi Volcanoes National Park (563) and Mauna Kea state lands (79) on Hawaiʻi, Haleakalā National Park (239) on Maui, and Kalaupapa National Historical Park (138) on Molokaʻi. Plot frequency chart x-axis values indicate the upper end of each category (e.g., 1000 = plots located 501–1000 m).

This study was conducted in the Hawaiian Islands, USA (19–22° N, 155–160° W; Fig 1). Sampling occurred in national parks on the islands of Hawaiʻi, Maui, and Molokaʻi and on state-protected lands at high elevations on Mauna Kea Volcano on Hawaiʻi. Sites ranged from the coast to 3000 m a.s.l.. Annual precipitation ranges from 500 to 10,200 mm with mean annual temperatures ranging from 7.7 to 23.5°C [43,44]. The Hawaiian Islands are volcanic in origin, and sampled substrates ranged from less than 400 years old on Hawaiʻi to over one million years old on Maui and Molokaʻi. Sampled plots were located across a wide range of soil types, including seven of the 12 known soil orders [45] and 45 different soil series [46,47]. Sampled plots were found on slopes angled up to 35 degrees, facing all aspects among the three islands.

Vegetation in the Hawaiian Islands has traditionally been classified based on elevation, moisture, [dry (< 1200 mm annual rainfall), mesic (1200–2500 mm), wet (> 2500 mm)] and physiognomy, based on the percentage cover of the dominant life form in the uppermost vegetation layer [35]. Our study area encompassed many of these combinations. Mueller-Dombois and Fosberg [48] combined these classification types into nine regional vegetation categories, seven of which were found within our study area: lowland dry forest, savanna, scrub, and grassland; lowland to upper montane seasonal forests; lowland to upper montane rain forests, including fernlands; montane cloud forests; montane bogs; high altitude vegetation; deserts and new vegetation on volcanic surfaces. Similarly, the study sites span fifteen Holdridge life zones, ranging from subtropical basal wet forest to subalpine boreal dry scrub [49]. Over 165 vegetation associations were described within our study sites [50,51,52,53]. Although all sites are within protected natural areas, non-native ungulates and plants have altered these communities to varying extents.

### Vegetation data

We used three vegetation data sets, totaling 1019 plant community plots (Table 2) and 617 plant species. Two data sets were collected by the National Park Service Pacific Island Inventory and Monitoring Program within Hawaiʻi Volcanoes, Haleakala, and Kalaupapa national parks on Hawaiʻi, Maui, and Molokai, respectively (archived by Pacific Island Inventory and Monitoring, Hawaii National Park, HI, USA). One data set contains 576 circular (400 m^2^) and 172 rectangular (700 m^2^) plots installed in homogeneous areas across each park (0–3000 m a.s.l.) for vascular plant inventories and classification of plant communities. The second contains 192 rectangular plots (1000 m^2^) installed in park wet forests and subalpine shrublands to assess status and detect long-term trends within upland plant communities. The third contains 79 high elevation (>2000 m) square plots (400 m^2^) on Mauna Kea (archived by United States Geological Survey, Hawaii National Park, HI, USA). These are the only plots not within a national park and were collected by the United States Geological Survey Biological Resource Division to assess habitat quality for an endangered Hawaiian honeycreeper bird (*Loxioides bailleui*)[54]. For these three data sets all plant species were recorded within each plot; plant nomenclature was standardized [37,55,56]. All necessary permits were obtained for the national parks vegetation data sets through the research permit program (HAVO-2010-SCI-0005, HAVO-2010-SCI-0011, HALE-2010-SCI-0007, HALE-2012-SCI-0001, KALA-2011-SCI-0008) and for the USGS vegetation data through the State of Hawaii Division of Forestry (see [54]).

**Table 2:** Three data sets used to rank plant species’ habitat specialization. Data were collected between 2004 and 2012 with plot size ranging from 400–1000 m^2^.

Study	Year	Plot Size	Island			Total
			Hawaiʻi	Maui	Molokai	
NPS I&M Vegetation Mapping Inventory	2005–2011	400 m^2^	461*	179	108	**748**
NPS I&M Plant Community Monitoring	2010–2012	1000 m^2^	102	60	30	**192**
USGS BRD Palila Habitat Survey	2004–2005	400 m^2^	79			**79**
**Total**			**642**	**239**	**138**	**1019**

*172 of these plots are 700m^2^ legacy plots from when Hawaiʻi Volcanoes NP acquired the Kahuku Unit [57].

Most of the sample plots are 400 m^2^ (64%); however, the larger (700–1000 m^2^) plots were retained for analysis to increase the diversity of habitats represented and better reflect species’ niche breadth. With these additional plots, increased replication and a greater species pool enabled more species to be ranked. Species richness was not corrected for the larger plots because (i) species habitat specialization rank order did not differ when the larger plots were excluded, (ii) local species richness had little influence on species rankings when using the multiplicative Jaccard index for beta diversity (see below), and (iii) previous studies found little effect of differing plot size on richness within this range of sizes, and no effect for analysis [16].

### Species habitat specialization

Plant species niche breadth or habitat specialization was estimated for each species based on patterns of co-occurrence with other species, using an algorithm originally proposed by Fridley et al. [16], with a multiplicative beta diversity modification as described by Manthey and Fridley [20]. This method is based on the assumption that species occurring across a range of habitat types are habitat generalists and have relatively high beta diversity (rate of species turnover among plots in which they occur). Alternatively, species with limited habitat preference are specialists which, regardless of their frequency in the data set, have relatively low beta diversity because they consistently occur with the same species. Presence/absence data were used for each plot.

Beta diversity was calculated as the pairwise Jaccard index to control for differences in species richness per plot among community types or regional species pool size.

Jaccardindex=(1‐j/(a+b‐j))

where *j* = the number of species found in both sites, *a* = the number of species in site A, and *b* = the number of species in site B [58]. For each plant species, all plots containing that species are compared pairwise to derive a mean Jaccard index for that species. This index equals 1 in cases of complete dissimilarity (i.e., no shared species among pairs of plots) in which case the species being assessed is interpreted as a strong habitat generalist. Alternatively, a value of 0 indicates complete similarity among plots (i.e., where every plot in which the species is found has identical co-occurring species) in which case the species is interpreted as a strong habitat specialist. However, in large regional scale data sets such as those used for co-occurrence analysis, species on the specialist end of the continuum are unlikely to be zero because of the non-uniform distribution of most plant species, small plot size, and the potential for observer error (particularly with cryptic species) just as actual values of generalists are unlikely to be one.

Controlling for abundance is also important, because species differ in frequency of occurrence in the data sets. Frequency may be due to the original site selection design and/or varying geographic ranges of different habitat types independent of the species’ habitat specialization. To prevent frequent or geographically widespread species from being erroneously classified as habitat generalists simply because they have a higher probability of occurring in more sample plots, we randomly selected 20 plots at a time containing a species to calculate the Jaccard beta diversity index. This procedure was then repeated 100 times to generate mean Jaccard estimates for each species and used as the measure of habitat specialization and corresponding standard deviation. This procedure was calculated for all species with 25 or more occurrences in the data set. Twenty-five plots represents a balance between the quantity of plants analyzed and confidence in the resulting index [16], regardless of how many occurrences each species had within the full data set. The output from this analysis provides a mean Jaccard index between 1 (generalist) and 0 (specialist), standard deviation, alpha diversity, and plot occurrence value (number of plots occupied) for each species in the data set that occurs in at least 25 plots (2.5% of the 1019 sample plots). Species found in fewer than 25 plots were not directly ranked for habitat specialization using this methodology owing to lack of sufficient replication in the data set.

We tested for significant correlations between co-occurrence generated Jaccard index values and species regional pool size (average species richness per plot) and species abundance (species occurrence frequency within the data set) using Pearson’s product-moment correlation.

### Environmental variables and species traits

To test for a relationship between the Jaccard specialization index and individual species traits, independent data were compiled for flowering plant species from Wagner and colleagues [37] and for pteridophytes from Palmer [55] (both updated from Wagner and colleagues [56]). Values of many variables were not available for all 170 plant species, but for most variables, over 70% of the species were included in analyses. Variable descriptions, values, and classes for all species are available in S1 Appendix. Some environmental and biogeographic variables (e.g., number of different moisture types a species is known to occur in, known elevation limits) are included to test the validity of this methodology in correctly identifying habitat generalists and specialists based on co-occurrence within the plot data set.

We tested for statistically significant relationships between plot-co-occurrence-generated Jaccard index values and known environmental, biogeographic, and species traits using three methods. We used a one-way analysis of variance to first determine if Jaccard index values differ among species biogeographic origin types as we hypothesized (e.g., endemic, indigenous, non-native). Because significant differences in average Jaccard index value were found between endemic and non-native plant species (see Results), we then controlled for biogeographic origin in all other variable testing. For categorical variables, we used two-way analysis of variance to determine if habitat specialization rankings (Jaccard index) differed among categories. For continuous variables, we developed linear models to examine if and to what extent habitat specialization (Jaccard index) differs across specific variable values. All statistical analyses for habitat specialization and variable testing analyses were carried out using R 2.11 software [59]. All figures are presented with co-occurrence generated Jaccard index values along the y-axis for consistency.

## Results

### Species habitat specialization rankings

**Fig 2:**
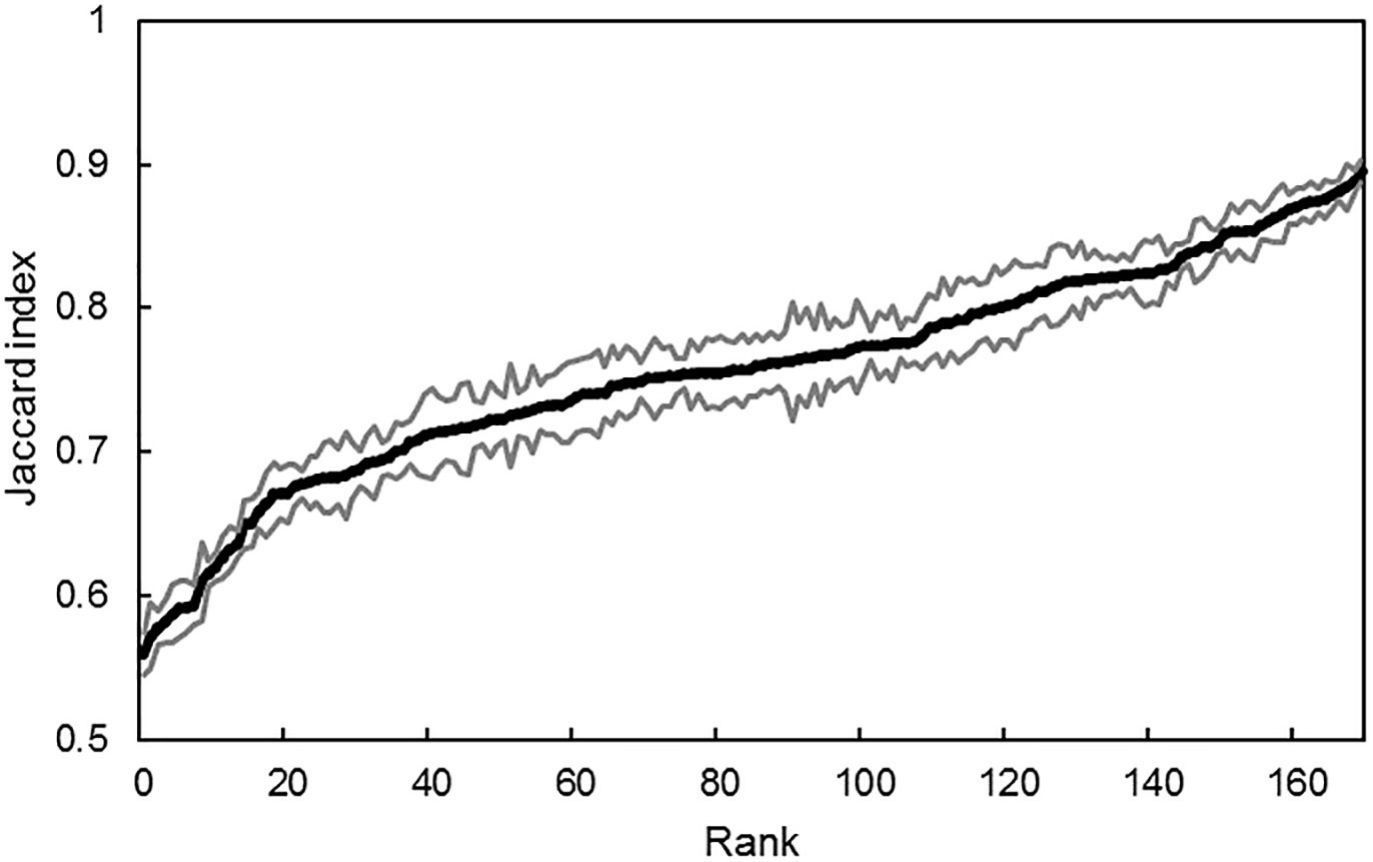
Habitat specialization (Jaccard index) and the standard error around the estimator for 170 plant species. The black center line indicates each plant species’ specialization index value (0 = complete specialist; 1 = complete generalist) and the grey lines are standard deviations. Species are ranked according to their specialization index.

Habitat specialization rankings for the 170 plant species that met the 25-plot occurrence cut-off (out of the total of 617 species encountered) are listed in S2 Appendix, from the most generalist species (*Oxalis corniculata* = 0.895) to the most specialist (*Crepis capillaris* = 0.560). Subsets including the twenty strongest generalists and specialists are provided in Table 3. The 170 ranked species represent 58 different plant families, 32 of which include exclusively native species and 13 of which include exclusively non-native species. Standard deviation per species was relatively consistent across the continuum with a mean of 0.02 (Fig 2).

**Table 3:** The twenty most generalist and most specialist plant species (highest and lowest Jaccard index values, respectively). The entire 170 plant species rank order and details are available in S2 Appendix. For each species, the Jaccard index value (standard deviation (SD) based on 100 permutations of selecting 25 plots), mean alpha richness per plot listed as μ(α), total number of plot occurrences out of 1019 possible, species origin, and life form are provided.

Species	Jaccard index ± SD	μ(α)	Occurrence	Origin	Life form
*Oxalis corniculata*	0.895±0.007	15.8	53	Non-native	Herb
*Ageratina riparia*	0.888±0.008	20.3	72	Non-native	Shrub
*Psidium cattleianum*	0.884±0.016	21.7	137	Non-native	Tree
*Conyza bonariensis*	0.882±0.007	18.6	29	Non-native	Herb
*Ageratum conyzoides*	0.879±0.009	13.3	31	Non-native	Herb
*Metrosideros polymorpha*	0.876±0.013	19.6	586	Endemic	Tree
*Schinus terebinthifolius*	0.875±0.009	14.2	52	Non-native	Tree
*Lantana camara*	0.874±0.014	12.1	75	Non-native	Shrub
*Ageratina adenophora*	0.873±0.011	24.8	50	Non-native	Shrub
*Psidium guajava*	0.871±0.012	13.8	64	Non-native	Shrub
*Cyclosorus dentatus*	0.869±0.010	18.2	38	Non-native	Fern
*Psilotum nudum*	0.866±0.020	24.8	66	Indigenous	Fern
*Nephrolepis brownii*	0.863±0.017	15.0	135	Non-native	Fern
*Ehrharta stipoides*	0.860±0.012	20.4	135	Non-native	Grass
*Emilia fosbergii*	0.857±0.010	14.8	25	Non-native	Herb
*Rubus rosifolius*	0.854±0.020	27.9	92	Non-native	Shrub
*Acacia koa*	0.854±0.020	22.2	110	Endemic	Tree
*Sphenomeris chinensis*	0.853±0.013	27.5	44	Indigenous	Fern
*Cenchrus clandestinus*	0.853±0.020	16.7	140	Non-native	Grass
*Rubus argutus*	0.852±0.011	19.7	45	Non-native	Shrub
*…*					
Middle 130 ranked plants (available S2 Appendix)					
*…*					
*Oenothera stricta*	0.671±0.017	16.3	26	Non-native	Herb
*Wikstroemia phillyreifolia*	0.671±0.022	12.9	34	Endemic	Shrub
*Festuca bromoides*	0.663±0.023	18.7	59	Non-native	Grass
*Diplopterygium pinnatum*	0.659±0.012	39.9	32	Endemic	Fern
*Rubus ellipticus*	0.651±0.017	34.2	29	Non-native	Shrub
*Nertera granadensis*	0.650±0.017	39.5	57	Indigenous	Herb
*Setaria palmifolia*	0.636±0.010	34.2	28	Non-native	Grass
*Asplenium normale*	0.633±0.016	37.8	32	Indigenous	Fern
*Labordia hedyosmifolia*	0.627±0.015	41.8	36	Endemic	Shrub
*Dubautia ciliolata*	0.620±0.010	13.0	30	Endemic	Shrub
*Medicago lupulina*	0.615±0.010	13.8	29	Non-native	Herb
*Thelypteris globulifera*	0.610±0.027	24.5	36	Endemic	Fern
*Heteropogon contortus*	0.593±0.014	11.1	33	Indigenous	Grass
*Dianthus armeria*	0.592±0.018	19.4	32	Non-native	Herb
*Hyparrhenia rufa*	0.591±0.020	9.8	41	Non-native	Grass
*Dubautia menziesii*	0.587±0.020	15.5	31	Endemic	Shrub
*Liparis hawaiensis*	0.582±0.015	36.9	26	Endemic	Herb
*Arrhenatherum elatius*	0.578±0.011	19.3	32	Non-native	Grass
*Geranium cuneatum*	0.572±0.023	16.2	40	Endemic	Shrub
*Crepis capillaris*	0.560±0.014	19.8	27	Non-native	Herb

**Fig 3:**
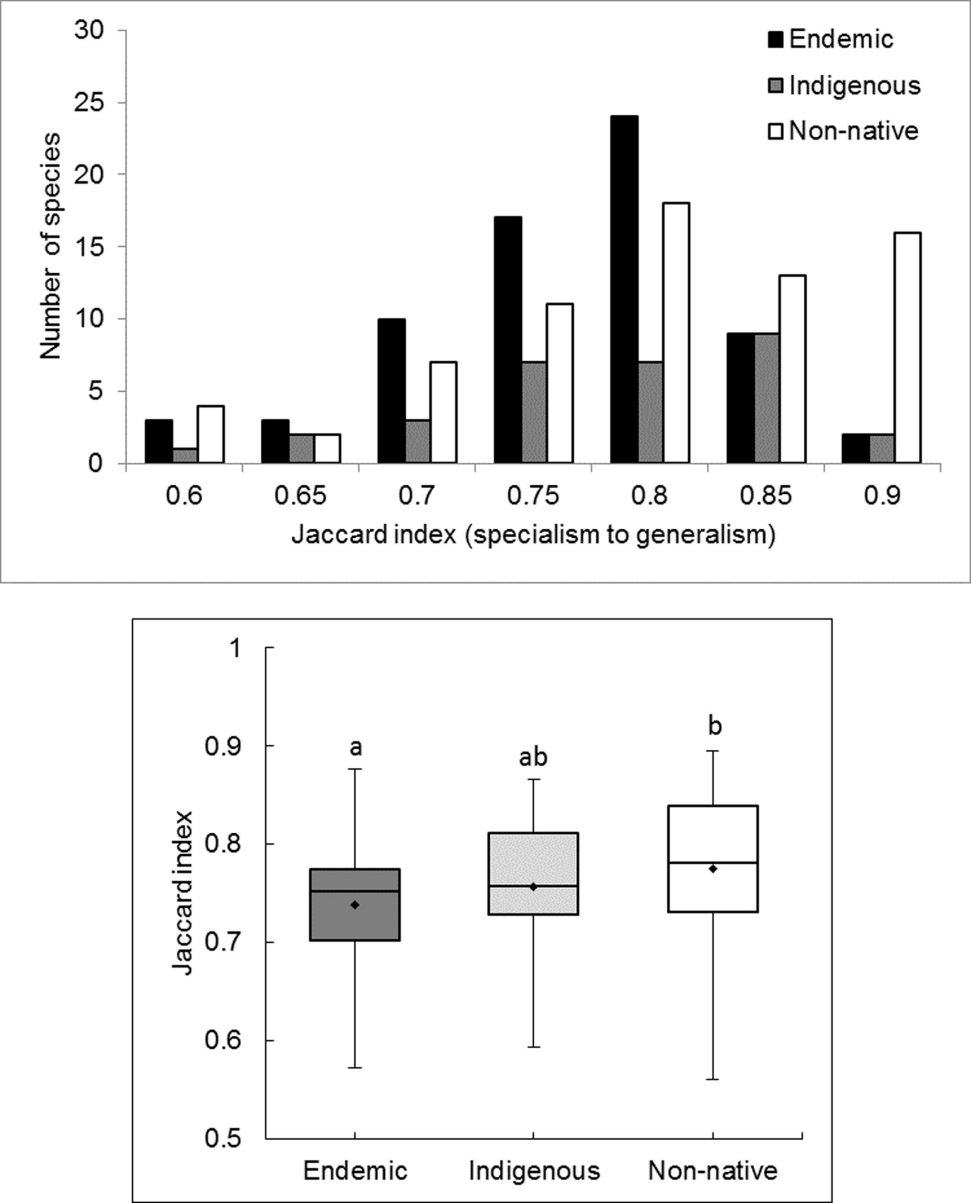
**A.** Histogram of species specialization by biogeographic origin. Specialization defined by the Jaccard index (0 = complete specialist; 1 = complete generalist) and biogeographic origin (endemic, indigenous, and non-native) for 170 plant species in the Hawaiian Islands. Percent of species by origin is presented to equalize categories. **B.** Box plot of species specialization by biogeographic origin. Non-native plant species (*n* = 71) are more generalized, with higher Jaccard index values, than endemic Hawaiian plant species (*n* = 68). Habitat specialization values for indigenous plant species (*n* = 31) are intermediate between endemic and non-native species. Diamonds within box plots are group means and lines are median values. Categories that share the same letter are not significantly different (*F*(2,167) = 4.735, *p* = 0.01).

The distribution of plant species differed by biogeographic origin (endemic, indigenous, non-native). Endemic Hawaiian plant species (*n* = 67) were more specialized than non-native plants (*n* = 71) and indigenous species (*n* = 32) were intermediate (Fig 3A and 3B). Representative endemic specialists tend to be restricted to high elevations, e.g., *Geranium cuneatum* shrubs and some *Dubautia* species (members of the silversword alliance adaptive radiation; [60]. However, two endemic tree species, *Metrosideros polymorpha* and *Acacia koa*, are ranked highly as generalists. Indigenous generalists include shrubs *Dodonaea viscosa* and *Leptecophylla tameiameiae* and pteridophytes *Sphenomeris chinensis* and *Psilotum nudum*, whereas indigenous specialists include species restricted to managed coastal landscapes, for example *Heteropogon contortus* [61], although there is still some question as to whether this grass is indigenous or introduced [37]; climatically wet region fern *Asplenium normale*; and boggy site herb *Nertera granadensis*. Non-native generalists include multiple life forms, representing various stages of invasion, including highly invasive trees and shrubs *Psidium cattleianum*, *Schinus terebinthifolius*, *Lantana camara*, and multiple herbs and ferns.

Mean species richness of the plots in which the 170 ranked plant species occurred was 23.3, and median was 19.8 (range 9.8 to 41.8), based on the full data set of 617 species. Habitat specialization rankings calculated using the Jaccard index are not consistently correlated with regional species pool size measured as mean plot richness. For most species (61%) average plot richness values are less than 26 species, and no significant correlation was detected between the Jaccard index and richness (*r* = 0.23). However, for species found above the 26 species richness threshold, a strong negative correlation existed between Jaccard index values and increasing average plot richness (*r* = -0.78; S3 Appendix). The negative correlation above the 26 species richness threshold may be explained by the increasing association and specialization of these species to the Hawaiian wet forest, the most diverse sampled community type within these data. Species abundance, measured as the number of plot occurrences per species, was significant but weakly (*r* = -0.28) correlated with the Jaccard index (S3 Appendix). On average, ranked species were found in 84 plots with a mode of 32 plots, but ranged from the minimum cut-off of 25 (*Emilia fosbergii*, *Paspalum dilatatum*) to 586 (*Metrosideros polymorpha*) plots.

These rankings were used to test the validity of this co-occurrence methodology with known species’ environmental and biogeographic ranges and to identify species’ traits that can be used to predict species location along the habitat specialization continuum as described in Table 1. Summary results are presented in Table 4 with detailed findings in the following results sections.

**Table 4:** Findings from tests to validate species habitat specialization rankings and efforts to identify the predictability of species’ traits along the habitat specialization continuum.

Type	Variable	Generalist Correlation	Predictions for species ranked as generalists:	Findings
Env	No. of habitat moisture types	+	will be found in more habitat moisture types, than those ranked as specialists	*p* < 0.001
Env	Moisture type*	mesic >wet >dry	will be found in habitats that do not require highly specialized physiological adaptations to survive	n.s.
Env	Elevation range	+	will be found across a greater range of elevations than specialists	n.s.
Env	Elevation minimum	-	will not be restricted to high elevations where specialized physiological adaptations are necessary to survive cool climates	*p* < 0.001
Env	Elevation maximum	+	will not be restricted to low elevations where specialized physiological adaptations are necessary to survive warm climates	n.s.
Biogeo	Species biogeographic origin*	non-native >indigenous >endemic	are more likely to have recently colonized new habitats (non-natives) or repeatedly colonized multiple habitats (indigenous) than species unique to a defined geographic location, such as an island (endemic)	*p* = 0.01
Biogeo	No. of Hawaiian Islands (1–6)*	+	are more likely to occur on multiple Hawaiian Islands because they can persist in multiple habitats, including diverse substrates	*p* < 0.001
Life hist	Leaf size variability	+	are more likely to have greater leaf size variability	n.s.
Life hist	Life form*	woody >herbaceous	are more likely to be woody than herbaceous life forms	*p* = 0.004
Life hist	Dispersal syndrome*	null model	will not differ from those ranked as specialists in seed dispersal syndrome (e.g., animal, wind, ballistic, gravity, no special adaption)	n.s.

*Categorical variables

### Testing the validity of habitat specialization rankings

**Fig 4:**
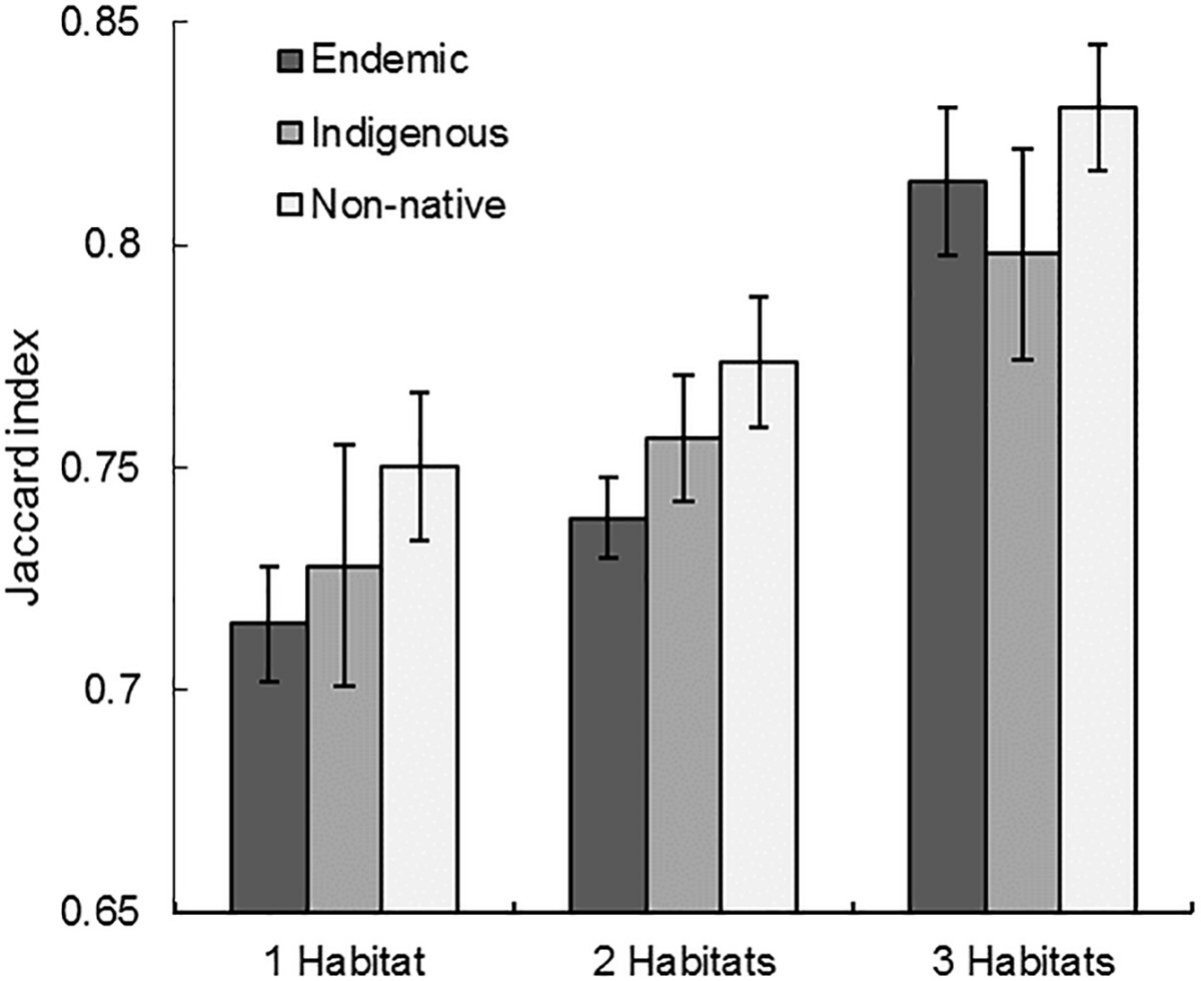
Species specialization by number of habitat moisture types. Plant species ranked as generalists (higher Jaccard index values) occur in more habitat moisture types (dry, mesic, wet) as hypothesized according to the published Hawaiian plant literature, than those limited to a single habitat moisture type (*p* < 0.001; Table 5). Mean values and standard errors are reported.

**Fig 5:**
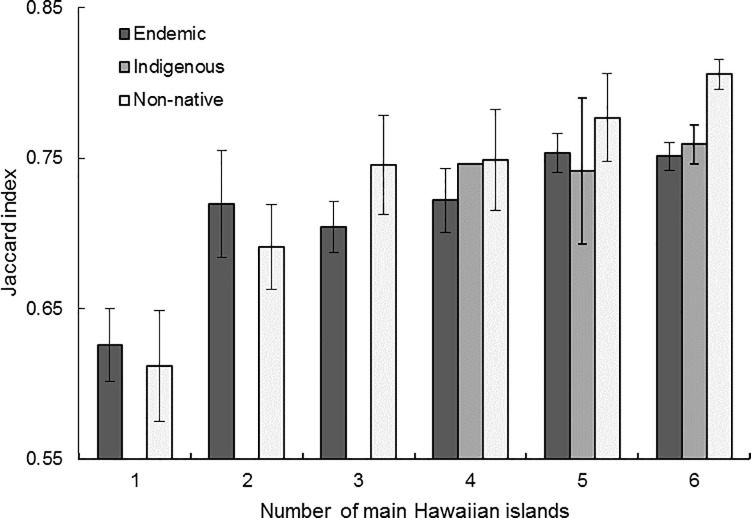
Species specialization by number of main Hawaiian Islands. Plant species ranked as generalists (higher Jaccard index values) occur on more Hawaiian Islands according to the flora, supporting the hypothesis that habitat generalists are more likely to occur on multiple islands because they can persist on diverse substrate types (*p* < 0.001; Table 5). Mean values and standard errors are reported.

**Fig 6:**
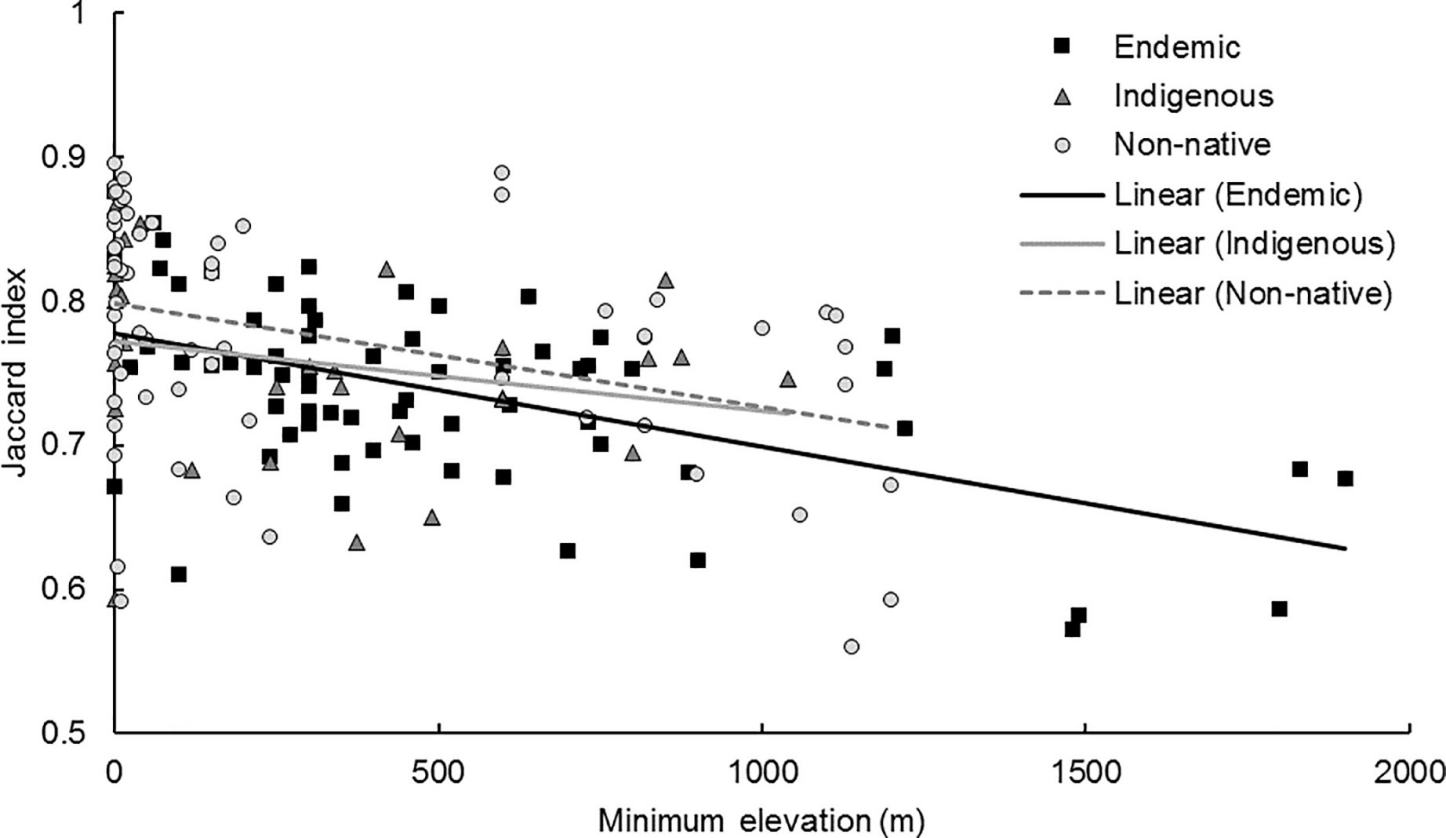
Species specialization by minimum elevation. A linear model was developed to determine if habitat specialization (Jaccard index) differs by minimum elevation and biogeographic origin. Plant species restricted to high elevations had lower Jaccard index values than those with low or no minimum elevation limit (*p* < 0.001; Table 6).

Habitat specialization rankings differed for three of the six tested environmental variables after controlling for differences in biogeographic origin: number of habitat moisture types (Table 5), number of Hawaiian Islands (Table 5), and minimum elevation (Table 6). As expected, increasing generalization, was associated with known species occurrence in multiple habitat moisture types (dry, mesic, and wet; Fig 4), supporting the validity of this method for using co-occurrence to estimate habitat niche breadth. However, the specific habitat moisture type(s) that species are known from was not related to differences in specialization. Increasing generalization was also associated with occurrence on more Hawaiian Islands (Fig 5), likely owing to the capacity of generalist species to persist on more diverse substrates. Increasing specialization was correlated with increasing minimum elevation (e.g., high minimum known elevation; Fig 6), which in turn was associated with decreasing elevation range for endemic and indigenous species (Table 6). However, because non-native species showed no relationship between specialization and elevation range, the resulting species specialization–elevation range linear model was weak (*R*^*2*^ = 0.10). Although many plants can be found at high elevations in the Hawaiian Islands, in our data set, only endemic species were restricted to these conditions. In contrast, no relationship was detected between species limited to low elevations (e.g., low maximum known elevation) and habitat specialization (Table 6).

**Table 5:** Two-way ANOVA’s. Conducted for each categorical variable (number of habitat moisture types, habitat moisture type, number of main Hawaiian Islands, life form, dispersal syndrome) to determine if habitat specialization rankings (Jaccard index) differed among categories while controlling for biogeographic origin.

Variable		SS	df	*F*	*p*
No. of habitat moisture types model					
	No. habitat moisture types (1–3)	0.121	2	12.78	**<0.001**
	Origin	0.038	2	4.06	**0.019**
	No. habitats moisture types x origin	0.003	4	0.16	0.958
	Residuals	0.763	161		
Habitat moisture type model					
	Habitat moisture type (mesic, wet, dry)	0.003	2	0.22	0.807
	Origin	0.013	2	1.05	0.358
	Habitat moisture type x origin	0.014	3	0.80	0.500
	Residuals	0.305	51		
No. of Hawaiian Islands model					
	No. of Hawaiian Islands (1–6)	0.207	5	9.84	**<0.001**
	Origin	0.058	2	6.83	**0.001**
	No. of Hawaiian Islands x origin	0.026	7	0.89	0.515
	Residuals	0.653	155		
Life form model					
	Life form (woody, herbaceous)	0.043	1	8.59	**0.004**
	Origin	0.071	2	7.02	**0.001**
	Life form x origin	0.018	2	1.81	0.168
	Residuals	0.825	164		
Dispersal syndrome model					
	Dispersal syndrome (bird, wind, other)	0.032	2	2.71	0.071
	Origin	0.049	2	4.09	**0.019**
	Dispersal syndrome x origin	0.030	4	1.27	0.287
	Residuals	0.667	113		

**Table 6:** Linear models. Developed to examine if and to what extent habitat specialization (Jaccard index) differs across continuous variables (elevation minimum, elevation maximum, elevation range, leaf size variance) and biogeographic origin. Models test the null hypotheses that (1) the Jaccard index is zero (Intercept = Endemic, Indigenous, Non-native) when controlling for the continuous variable and (2) that there is no relationship (slope = 0) between the Jaccard index and the continuous variable. Bold *p* values indicate significant differences from the null hypotheses. The minimum elevation and elevation range models had significant slopes; however, only the minimum elevation model explained greater than twenty percent of the variance in the data (*R*^*2*^) with endemic species displaying the steepest slope (Fig 6).

Variable		Est	SE	*t*	*p*
Minimum elevation x origin					
	Intercept	7.77e-01	1.22e-02	63.63	**<0.001**
	Indigenous	-5.21e-03	2.04e-02	-0.26	0.799
	Non-native	2.06e-02	1.61e-02	1.28	0.203
	Min elevation	-7.85e-05	1.81e-05	-4.34	**<0.001**
	Min elevation x indigenous	2.99e-05	4.09e-05	0.73	0.464
	Min elevation x non-native	6.53e-06	2.68e-05	0.24	0.808
	Residuals		6.53e-02		
	Model output	*F*(5,154) = 8.856, *p* = 2.08e-07, *R*^*2*^ = 0.22			
Maximum elevation x origin					
	Intercept	7.37e-01	3.10e-02	23.77	**<0.001**
	Indigenous	-1.67e-02	5.00e-02	-0.33	0.739
	Non-native	7.69e-02	3.91e-02	1.97	0.051
	Max elevation	3.14e-07	1.37e-05	0.02	0.982
	Max elevation x indigenous	1.83e-05	2.31e-05	0.79	0.430
	Max elevation x non-native	-2.30e-05	1.88e-05	-1.22	0.224
	Residuals		7.16e-02		
	Model output	*F*(5,154) = 2.589, *p* = 0.028, *R*^*2*^ = 0.08			
Elevation range x origin					
	Intercept	6.56e-01	2.64e-02	24.83	**<0.001**
	Indigenous	5.32e-02	4.20e-02	1.27	0.207
	Non-native	1.14e-01	3.38e-02	3.36	**0.001**
	Elevation range	4.89e-05	1.51e-05	3.24	**0.002**
	Elevation range x indigenous	-2.07e-05	2.34e-05	-0.89	0.378
	Elevation range x non-native	-4.53e-05	2.05e-05	-2.22	**0.028**
	Residuals		6.96e-02		
	Model output	*F*(5,154) = 4.492, *p* = 7.54e-04, *R*^*2*^ = 0.13			
Leaf size variance x origin					
	Intercept	7.25e-01	1.51e-02	47.96	**<0.001**
	Indigenous	2.35e-02	3.22e-02	0.73	0.467
	Non-native	6.70e-02	1.92e-02	3.49	**<0.001**
	Leaf size variance	6.34e-04	4.64e-04	1.37	0.174
	Leaf size var. x indigenous	-4.06e-04	1.44e-04	-0.28	0.778
	Leaf size var. x non-native	-1.49e-03	6.07e-04	-2.45	**0.016**
	Residuals		7.57e-02		
	Model output	*F*(5,111) = 2.685, *p* = 0.025, *R*^*2*^ = 0.11			

### Species traits used to predict habitat specialization

**Fig 7:**
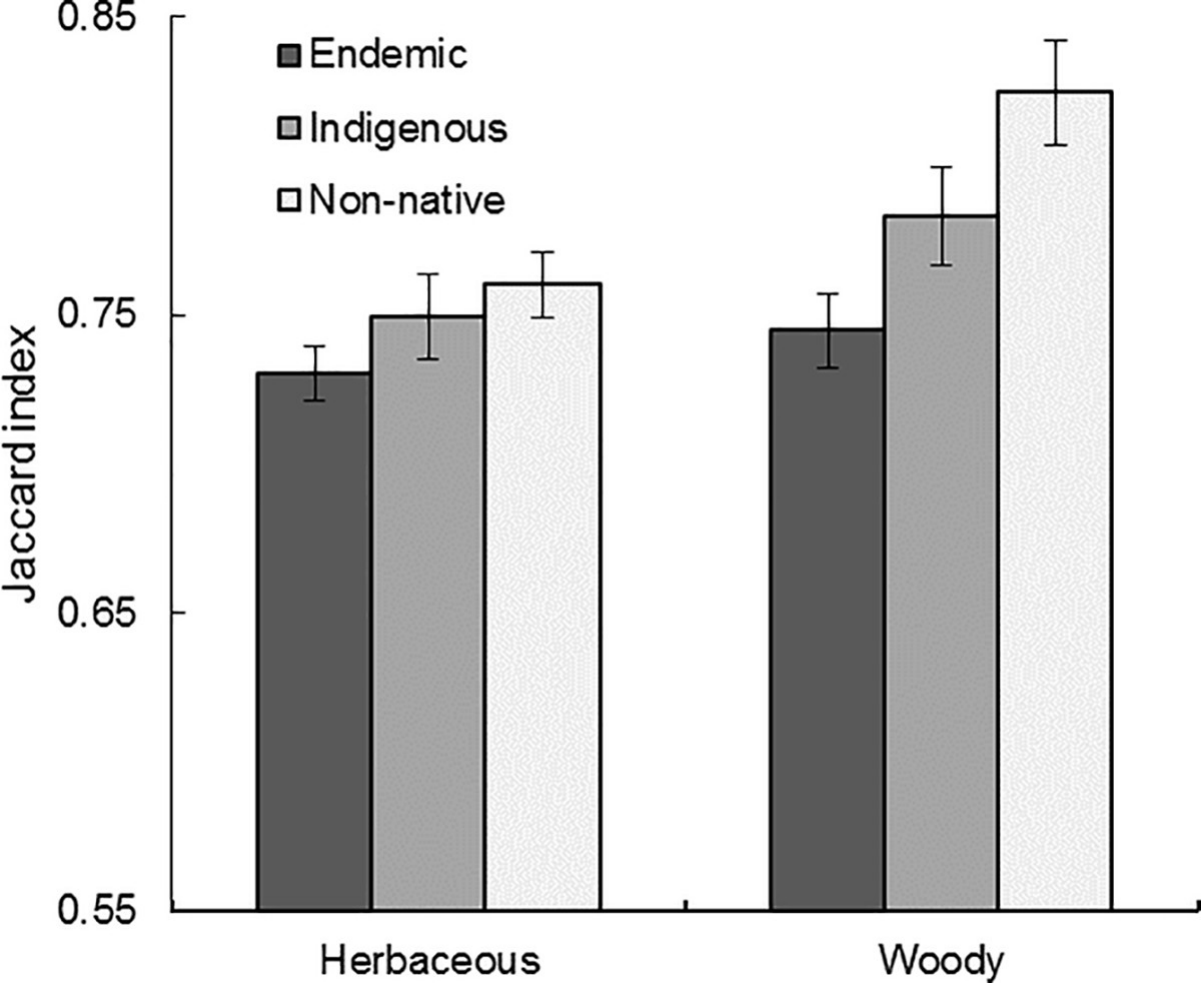
Species specialization of life form. Species habitat specialization differs between herbaceous and woody plant life forms (*p* = 0.004) when controlling for biogeographic origin (Table 5). This pattern is most evident in non-native species. Mean values and standard errors are reported.

**Fig 8:**
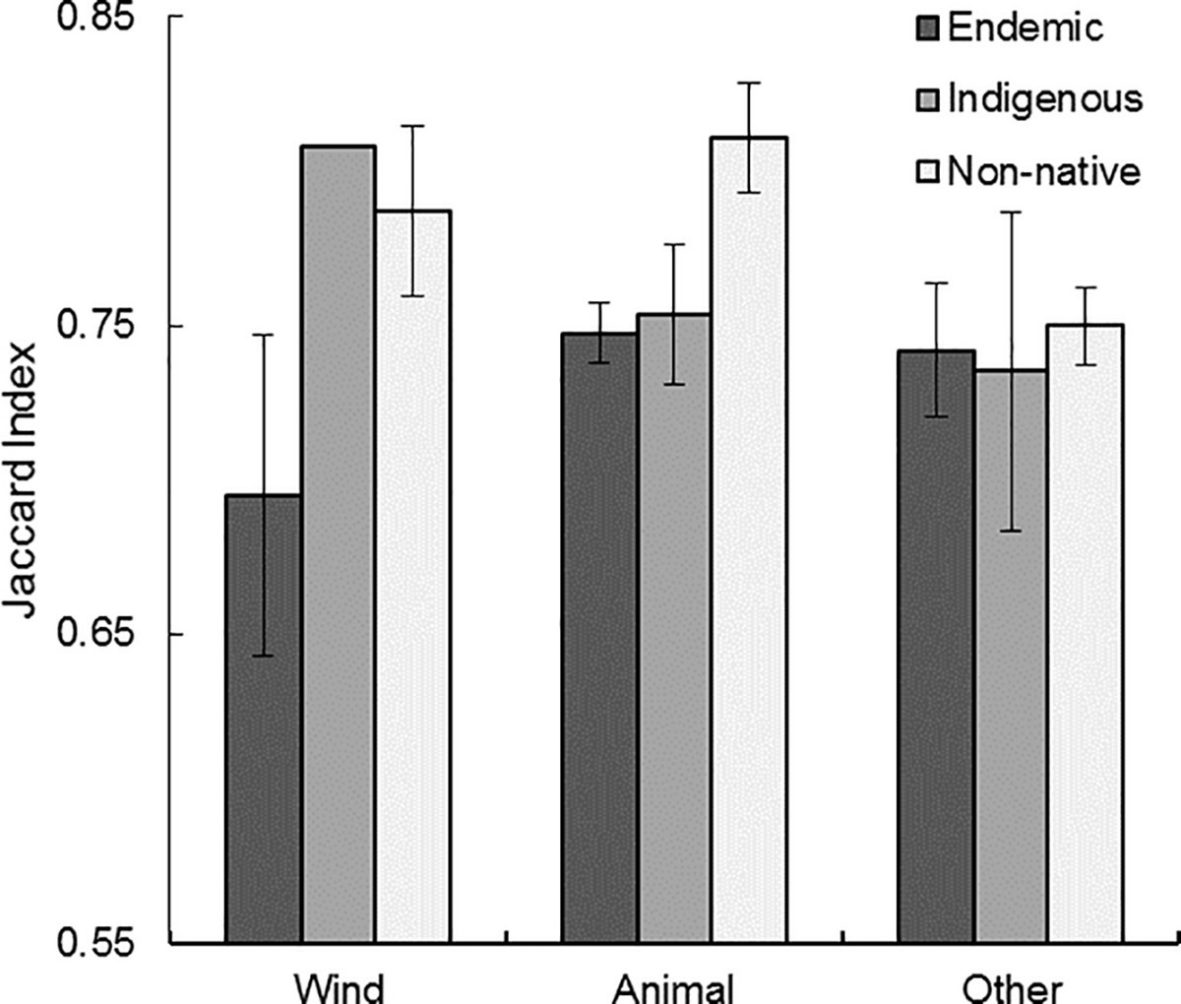
Species specialization by dispersal syndrome. As hypothesized, no significant difference was detected among dispersal syndromes across the specialization continuum (*p* = 0.071) when controlling for differences among biogeographic origin (Table 5). Mean values and standard errors are reported.

Life form was the only trait of the three tested that differed across the specialization continuum, with increasing generalization associated with woody species (Table 5). This pattern was particularly evident for non-native plant species (Fig 7), but this statistical trend is not strict, considering that the most generalized species in the data set is a non-native herb (Table 3). As hypothesized, no significant difference was detected among dispersal syndromes across the specialization continuum (Table 5) after controlling for differences among biogeographic origin (Fig 8). Contrary to our hypothesis, leaf size variance was important in explaining species specialization only for non-native plants, and the relationship was the opposite of our expectation (Table 6). Non-native plant species displayed a negative relationship between habitat generalization and leaf size variance.

## Discussion

### Species habitat specialization rankings

The relatively new, indirect approach of estimating habitat niche breadth from species co-occurrence data appears to be useful for classifying plants along a habitat generalist-specialist continuum. This application of co-occurrence data has been recognized as an efficient, quantitative estimate of habitat specialization which can be used to identify species threatened by rapidly shifting climates [62,11,63,64]. While loss of any native species is a global concern, loss of habitat specialists is likely to occur sooner and more quickly, implying that more immediate management attention is needed to monitor population changes [65]. As the proportion of generalist species increases within an ecosystem, it is likely that functional diversity decreases [65].

### Testing the validity of habitat specialization rankings

The distribution of plant species along the habitat specialization continuum differed by species biogeographic origin (endemic, indigenous, non-native) with endemic Hawaiian plant species ranked on the specialist end and non-native plant species ranked on the generalist end of the continuum. We expected and controlled for differences in biogeographical origin among species when examining if habitat specialization rankings differ by environmental variables.

#### Habitat moisture type

Species ranked as generalists were independently documented in the published Manual of the flowering plants of Hawaii [37] and Ferns of Hawaii [55] to occur in all three habitat moisture types (dry, mesic, and wet), supporting the validity of this co-occurrence-based habitat-niche-breadth ranking method. Contrary to our expectation, we did not find any evidence supporting the coarse environmental (e.g., temperature and/or moisture) harshness hypothesis [22,23,24], that species known only from dry (less productive) habitats are more specialized than those known only from mesic or wet habitats. This may simply be the result of specialists from diverse climatic conditions (e.g., dry cold sites vs. dry warm sites) canceling each other out and/or insufficient sample size. Endemic Hawaiian plants currently at risk, tend to have low historic population densities and are typically limited to specific habitat types. Habitat types range across a wide variety of abiotic conditions (e.g., low elevation dry environments, mesic lowland, montane forests, and wet montane forests), each with unique rare endemic plant species [66]. Fewer than 35% of the 170 species tested are known from only one habitat type, resulting in small sample size for comparison of specialization. It is likely that very rare species and species limited to one, narrow habitat type are underrepresented in our analysis, because we excluded those found in fewer than 25 plots (but see [67]). This study, like other tropical community analyses in diverse sites, would benefit from additional plot replication [68]. Additionally, considering the extensive history of anthropogenic disturbance in the Hawaiian Islands (e.g., agriculture, feral ungulate introductions, wildfire), some plants may rank as specialists due to habitat loss and some habitat specialists may have already been eliminated, since specialists tend to benefit from stable conditions with low disturbance intensity [69].

#### Elevation

Species ranked as specialists were more likely to be restricted to high elevations with harsher environmental conditions than species capable of persisting in lower, warmer habitats [70]. For example on Hawaiʻi and Maui Islands, the subalpine zone above the trade wind inversion layer (>1800m a.s.l.), is very dry and can be cold, coupled with high solar radiation [44], requiring specialist species to possess stress-tolerant adaptations [71]. Endemic *Geranium cuneatum*, which is restricted to these high elevation zones, has silvery pubescent leaves which likely provide diurnal solar protection, nocturnal cool temperature protection, and high water use efficiency. In our study, only endemic Hawaiian plants were restricted to higher elevations (>1200 m a.s.l.), reflecting the current understanding that some if not many high elevation endemic Hawaiian plant species derive from high elevation temperate ancestors [72,73,74]. In contrast, non-native invaders tend to arrive on oceanic islands via lowland introduction pathways [75,76]. Introduction patterns for non-native plant species strongly favor human transport routes which were historically by sea but now include an exceptional diversity of routes from expanding global transport networks [77,78]. When comparing mountains from around the world, proximal lowland non-native floras were the main determinant of mountains’ non-native species composition [79], and few invaders were highly specialized stress-tolerant species; rather, invaders tended to have wide climatic tolerances [76]. Similarly, in Hawaiʻi, numerous non-native plant species, often of European origin, are found both in harsh subalpine sites, as well as in disturbed warmer sites at lower elevations. Non-native species typically found at high elevations (e.g., *Holcus lanatus*, *Hypochoeris radicata*) tend be from temperate regions, possibly owing to climatic conditions at higher elevations that favor cold tolerance. Introduced temperate species are primarily herbaceous and were facilitated by human land use directly (e.g., fodder introduced for ranching) and indirectly (e.g., plants attached to introduced fauna) [80]. High elevation sites are under increasing invasion pressure with increasing tourism, propagule pressure, introductions of mountain specialists’ species for horticulture, and climate change [81], which could increasingly threaten endemic Hawaiian high elevation specialists.

We expected species with narrow known elevation ranges to be specialists, but our model for elevation range explained little variance. This lack of correlation between elevation envelope and specialization may be a byproduct of the values originating from the Manual of the flowering plants of Hawaii [37]. These ranges are “imperfectly understood” because values were obtained from incomplete herbarium records with few detailed distributional studies. Most species (87%) ranked in this study had very broad (>1000 m) and overlapping elevation ranges, resulting in high model variance. Alternatively, broad overlapping ranges may be an accurate representation of non-rare plant species distributions in Hawaiʻi. Kitayama [36] found much lower beta diversity on Haleakala, Hawaiʻi as compared to Mount Kinabalu, Borneo, a continental island with comparable age, climate, and generic diversity. Presumably Hawaiʻi’s extreme isolation resulted in initial floristic impoverishment resulting in few species with very wide environmental niche breadths. Considering the abundance of native species with wide environmental niches, Hawaiian plants may fare better than their continental relatives with shifting climatic conditions.

Maximum elevation was not a significant predictor of Jaccard’s habitat specialization index, indicating that our sampled species are not restricted to warm or coastal environments. Previous research has identified a distinct suite of primarily indigenous and some endemic species in Hawaiʻi restricted to the coastal strand and salt spray environments [82], but our data set was too coarse to capture an adequate sample of species (1%) restricted to lowland environments (<500m).

#### Number of Hawaiian Islands

Species ranked as generalists tended to occur on more Hawaiian Islands than those ranked on the specialist end of the continuum. This pattern maybe due to generalist species’ capacity to persist across more diverse substrates. The presence of plant species on multiple islands is evidence of long-distance dispersal (natural or anthropogenic) and a lack of subsequent evolutionary divergence. As predicted, this pattern was strongest among non-native plant species, which by their presence in Hawaiʻi demonstrate anthropogenic long-distance dispersal capabilities and limited time for divergence. Many of the same non-native plant species are found on islands around the world, presumably owing to recurrent human introductions; whether they become invasive depends on additional variables (e.g., time lags, habitat type invaded, biotic and abiotic factors; [83]). Occupancy or residence time for non-native species is important for several reasons: time is necessary for spread, niche breadth of invaders may expand over time owing to increased dispersal and/or adaptation, time increases the likelihood of favorable stochastic events, and even abiotic variables associated with climate change in ecological time may provide additional opportunities for spread [81]. In our data, time since the introduction of non-native species [37] was positively correlated with habitat generalism (*r* = 0.45, *p* < 0.001), suggesting that the longer non-native species have been in Hawaii, the more diverse habitats they colonize. For multiple life forms (e.g., fern, herb, shrub, tree), the strongest non-native generalists were documented as present before the early 1900’s. *Cyclosorus dentatus* (= *Christella dentata*), native to the tropical/subtropical Old World and widely invasive in the Americas, was initially collected on Oahu in 1887. This terrestrial fern was the first non-native fern reported as naturalized in Hawaiʻi [55]. The widespread herb, *Oxalis corniculata*, is a cosmopolitan species of unknown origin and has been suggested as possibly a Polynesian introduction or even an indigenous species, because it was collected in Hawaiʻi in 1779 by David Nelson, the first botanist to visit the Islands [37]. *Ageratina riparia*, a sprawling subshrub, is native to Mexico and the West Indies and has been present since before 1926 [37]. These species were likely accidental introductions, with the possible exception of *O*. *corniculata*, and are now widespread across the islands, but generally not of great conservation or agricultural concern with one noted exception. *Ageratina* was a major agricultural pest in the Hawaiian Islands prior to the introduction of several biological control agents in the 1970’s which quickly controlled the herb in rangelands across the islands [84]. The strongest generalist non-native tree species, *Psidium cattleianum*, native to the Neotropics, is believed to have first been intentionally introduced in 1825 and is now considered one of the most serious invasive plants in the Islands [37,85].

Many non-native plant species in Hawaiʻi are likely stronger generalists than our data indicate, because they may not have reached their full invasion potential. Little evidence of rapid genetic adaptation has been found in Hawaiian invasive species (e.g., *Clidemia hirta* [86]); however, only a few invasive have been studied in this regard. Some of the herbs and grasses classified on the specialist end of our continuum are either recent invaders or documented as rapidly spreading. The perennial grass, *Arrhenatherum elatius*, previously known from a single collection in Hawaiʻi since 1936 [37], is now considered naturalized in East Maui [56]. The broadleaved invasive grass *Setaria palmifolia* first documented in Hawaiʻi in 1903 [37], has become increasingly widespread within Hawaiʻi Volcanoes National Park during the past fifty years [57] and was recently documented for the first time on Kauaʻi and Molokaʻi [56]. The mean time since introduction for the 25 strongest non-native specialists (93 years) is significantly shorter than the mean time since introduction for the 25 strongest non-native generalists (125 years) in this study (*t* = 3.085, df = 29, *p* = 0.004). The rapid rate of introduction and naturalization of new species (see numerous newly naturalized plant species in Wagner [56]) suggests that there is a continuous supply of new species positioned to take advantage of the increasing disturbance that is forecast to coincide with climate change [87]. However, we do not expect that all new invaders will eventually function as generalists in Hawaiʻi, because many of the more recent introductions are for horticulture and may require more specialized habitats as opposed to the past widespread global accidental introductions [88].

Like non-native species, habitat specialization of endemic Hawaiian plants is correlated with number of islands occupied. Although some single-island endemics are present (e.g., *Dubautia ciliolata* and *Wikstroemia phillyreifolia* on Hawaiʻi, *D*. *menziesii* on Maui), most endemic species in our data set are found on multiple islands. Habitat specialization was not correlated with number of islands for the 32 indigenous plant species we ranked; however, all these species were present on four or more of the main islands.

### Species traits used to predict habitat specialization

Species traits are increasingly used by ecologists to forecast effects of climate change on future biodiversity [89] because they are easier to quantify than system processes, and traits can depict tradeoff patterns between plant’s allocation to photosynthesis/growth and storage/defense consistently across climatic gradients [90].

#### Life form

Life form was the only trait that differed across the specialization continuum, with woody species ranked as stronger generalists than most herbaceous species. This pattern was particularly evident for non-native plant species. Although globally, herbaceous life forms, specifically grasses, are found within nearly all biomes [91], the individual woody species in our study co-occurred with a greater diversity of species than the individual herbaceous species suggesting that woody species may often be better equipped to survive with shifting climatic conditions. Alternatively, it is possible that herbaceous species were missed during sampling more than woody species due to their more ephemeral nature and/or observer error.

#### Leaf size

Leaf size variability did not differ significantly across the habitat specialization continuum. Our sample size was reduced by nearly one-third for this analysis because pteridophytes lacked frond size data. Additionally, leaf size does not capture thickness or mass and therefore does not necessarily function as a surrogate for the widely recognized functional trait, specific leaf area (SLA). SLA is frequently used as a positive measure of plant growth and efficiency: plants with higher SLA tend to be faster growing [92]. We expected that habitat specialists would have less leaf size variability due to the physiological challenges of persisting in harsher environments, but one global review found only a modest effect of climate on leaf trait relationships [93]. Further, in mixed broad leaved mesic deciduous forests in Slovenia, habitat specialists had significantly higher SLA than generalists [70]. This highlights a potential limitation of using traits as indicators of habitat specialization and/or indicators of climatic flexibility in the face of climate change. Habitat specialists are specialized to specific environmental conditions which may lead to conflicting species trait adaptations when traits are averaged across large scales. For example, in our study, specialists range from native endemic species adapted to the dry, cold, high elevation subalpine habitats (e.g., *Geranium cuneatum*) with specific adaptations (i.e., small silver pubescent thick leaves for diurnal solar protection, nocturnal cool temperature protection, and water efficiency), to non-native invasive African C4 perennial grasses dominating the dry, hot, coastal lowlands (e.g., *Hyparrhenia rufa*) with quite different specific adaptations (i.e., long thin leaves and underground root mass to survive repeated fire) to persist with regular anthropogenic disturbance and biotic homogenization [65,24]. Taken together, mean leaf size values for these specialists may be misleading because the grass leaves are six times larger than the high elevation herb leaves.

#### Dispersal mechanism

As hypothesized, dispersal mechanisms of generalist and specialist species did not differ in our study. Previous studies identified relationships between bird dispersal in North American trees and habitat generalist species [16] and/or rapid tree migration following the last ice age [34,94]. Fridley and colleagues [16] found animal dispersal to be positively correlated with habitat generalists; however, many of the specialist trees in their study are in the Pinaceae and have known animal dispersal mechanisms. Further, caution is necessary when applying patterns of rapid plant migration from the early Holocene to current climate change predictions, because today’s landscape is far more fragmented [95]. In Hawaiʻi, land area is extremely limited, and very long-distance dispersal for plants may not be necessary for establishment. The dominant canopy tree, *Metrosideros*, is wind dispersed, yet because it is so abundant, it is not thought to be dispersal limited at least on Hawaiʻi Island [96].

Although previous studies in Hawaiʻi have highlighted inherent differences in functional traits between native and non-native species [97], studies elsewhere found no systematic differences in traits or colonizing abilities between native and non-native species [98,99]. In our study, we had data to test only three basic traits (life form, leaf size, and dispersal) with limitations previously highlighted for each. More functional trait data (e.g., wood density, leaf traits, pollination, seed size) are needed for Hawaiʻi to determine which traits, if any, are predictive for a species’ position along the specialist-generalist continuum. Recent studies have begun to collect additional trait data [100,101,102], but they are limited to few species and/or specific habitats on single islands as opposed to capturing the range of trait plasticity for multiple species. When using species functional traits, we are assuming that interspecific variability is greater than intraspecific variability [103,31] which may not be valid for many of Hawaiʻi’s plant species with their remarkably wide ecological amplitudes.

## Conclusions

In this study, we have shown that quantifying species habitat specialization using a similarity index derived from co-occurrence plot data appears to be valid, as the environmental and biogeographical relationships were consistent with theoretical expectations (e.g., generalization associated with occurrence in more habitat moisture types and diversity of substrates among islands). This indirect method of estimating habitat niche breadth uses increasingly available, large, plant community data sets with output rankings which represent species’ realized niches. In addition to species’ physiological limitations, it is important to capture the effect of biotic mechanisms on species distribution in Hawaiʻi, as this co-occurrence method does, because of the strong influence of non-native plants and animals in most communities. Additionally, the physiological limits that define a species’ fundamental niche are not known for most species and are unlikely to be known in time to manage them in the face of rapidly shifting climates [17].

We found limitations in translating Manthey and Fridley’s [20] Jaccard index to species’ traits. Predictions were limited by the paucity of available trait data for Hawaiian plant species and appropriate trait ranges for non-native species within the Hawaiian Islands. Theoretically, as functional trait data become available, this methodology can identify predictor traits of habitat specialization. However, it is still unclear whether our lack of significant correlations between specialization and species’ traits is simply a result of data limitations, or rather is expected because highly variable specialist trait values across the range of contrasting harsh environments in Hawaiʻi become balanced [104], and/or because of Hawaiʻi’s disharmonic flora and high endemism.

Even with these limitations, our data support the need for careful monitoring of native Hawaiian habitat specialists, so declines can be detected quickly. Consistent with other insular environments, many invasive non-native species in Hawaiʻi are more generalized and therefore likely to continue benefiting from additional climatic changes, as long as change does not go beyond their climatic envelope, at the expense of native specialists. Non-native woody species as a group are the strongest generalists in our data, supporting the long-appreciated management perception in Hawaiʻi that woody invaders represent a greater threat than herbaceous invaders to native species conservation. Our findings highlight the importance of conserving high elevation plant communities in the islands. These relatively intact plant communities may well be facing the greatest threat from the synergy of increased non-native plant propagule pressure, expanding human visitation, and shifting climatic conditions. Although our study does not address genetic adaptation, it is unlikely that high elevation endemic specialists will be capable of adapting to shifting climatic conditions because these extreme marginal habitats typically represent the edge (= physiological limits) of species populations [8]. Greater conservation attention must be focused on these high elevation plant communities, which have already experienced the greatest change in climatic conditions [39] in Hawai‘i. Additionally, high elevation communities represent some of the only remaining vegetated refugia for Hawaiian forest birds threatened by mosquito-vectored avian malaria [105,106].

The management implications of this study highlight the urgent need to identify and monitor species and their plant communities that are on the specialist end of the continuum. Globally, the loss of habitat specialists is a clear indication of ongoing degradation [11]. This technique allows us to identify species that are currently common or even dominant and fall within the specialist range, such as the endemic shrub *Dubautia menziesii*. Such common specialist species are of concern because common species are often overlooked in favor of the many threatened and endangered species in Hawaiʻi. Even globally, common species are often overlooked, even though systemic declines associated with common species are increasingly recognized in a variety of taxa and the loss of naturally occurring common species is bound to be devastating to ecosystem structure, function, and services [107,108]. Common species on the specialist end of the continuum should be targeted for additional climate change mitigation research (e.g., physiological sensitivity analysis) to support natural area managers in the struggle to preserve and conserve biological diversity in the face of climate change.

## Supporting information

S1 Appendix Plant species’ environmental and biogeographic known ranges, and predictive traits. (DOCX)

S2 Appendix Habitat specialization rankings for the 170 plant species that met the 25-plot occurrence cut-off. (DOCX)

S3 Appendix Relationship between habitat specialization rankings and species richness and species occurrence within plot data. (DOCX)
