# Supplementary material for: Classifying Hawaiian plant species along a habitat generalist-specialist continuum: Implications for species conservation under climate change
Source: PLoS One. 2020 Feb 7;15(2):e0228573. doi: 10.1371/journal.pone.0228573 (PMC7006925; doi:10.1371/journal.pone.0228573)
Supplement: S1 Appendix — (DOCX) [file pone.0228573.s001.docx]

**S1 Appendix. Plant species’ environmental and biogeographic known ranges, and predictive traits.**

Species known environmental and biogeographic ranges as well as trait values were derived from the *Manual of flowering plants of Hawaii* (Wagner et al. 1999), *Hawaii’s ferns and fern allies* (Palmer 2003), and updates to both references in *Hawaiian vascular plant updates* (Wagner et al. 2012). Biogeographic origin is abbreviated as endemic (End), indigenous (Ind), and non-native or naturalized (Non). Life form is presented as five categories for additional detail; however for analysis life form is categorized as woody (Tree or Shrub) versus herbaceous (Herb, Fern, Grass). Habitat is summarized as dry (D), mesic (M), and wet (W). Main Hawaiian Islands include Hawaiʻi (H), Kauaʻi (K), Lānaʻi (L), Maui (M), Molokaʻi (Mo), and Oʻahu (O). Dispersal syndrome is simplified into three categories with ferns excluded. Elevation values exclude extreme outliers and elevation range is calculated as maximum minus minimum elevation. Leaf size variance is calculated as the maximum area (length x width) relativized by the minimum leaf area to account for different size leaves and leaflets. Ferns were excluded from leaf size variance analysis. Blank cells indicate missing data and are not included in analysis.

| **Species** | **Origin** | **Life form** | **Habitat** | **Main islands** | **Dispersal syndrome** | **Min. elev. (m)** | **Max. elev. (m)** | **Leaf size variance (cm^2)** |
| --- | --- | --- | --- | --- | --- | --- | --- | --- |
| *Acacia koa* | End | Tree | D,M,W | H,K,L,M,Mo,O | Other | 60 | 2060 | 17.3 |
| *Adenophorus hymenophylloides* | End | Fern | W | H,K,L,M,Mo,O |  | 350 | 1750 |  |
| *Adenophorus pinnatifidus* | End | Fern | M,W | H,K,L,M,Mo,O |  | 240 | 1680 |  |
| *Adenophorus tamariscinus* | End | Fern | M,W | H,K,L,M,Mo,O |  | 300 | 1300 |  |
| *Adenophorus tenellus* | End | Fern | M,W | H,K,L,M,Mo,O |  | 365 | 1675 |  |
| *Adenophorus tripinnatifidus* | End | Fern | M,W | H,K,L,M,Mo,O |  | 600 | 1950 |  |
| *Ageratina adenophora* | Non | Shrub | D,M,W | L,M,Mo,O | Wind | 600 | 2000 | 22.5 |
| *Ageratina riparia* | Non | Shrub | D,M,W | H,K,L,M,Mo,O | Wind | 600 | 2000 | 3.0 |
| *Ageratum conyzoides* | Non | Herb | D,M,W | H,K,L,M,Mo,O | Wind | 0 | 1300 | 7.8 |
| *Agrostis sandwicensis* | End | Grass | D | H,K,M,O, | Other | 1190 | 3540 | 4.0 |
| *Alyxia stellata* | End | Shrub | D,M,W | H,K,L,M,Mo,O | Bird | 50 | 2000 | 37.5 |
| *Anagallis arvensis* | Non | Herb | D,M | H,K,L,M,Mo,O | Other | 0 | 2450 | 8.8 |
| *Andropogon virginicus* | Non | Grass | D,M | H,K,L,M,Mo,O | Other | 50 | 1200 | 4.4 |
| *Anthoxanthum odoratum* | Non | Grass | W | H,K,M,Mo,O | Other | 840 | 2140 | 14.0 |
| *Arrhenatherum elatius* | Non | Grass | M | H,M | Other |  |  |  |
| *Asplenium adiantum-nigrum* | Ind | Fern | D | H,K,L,M,Mo,O |  | 350 | 4000 |  |
| *Asplenium contiguum* | End | Fern | M,W | H,K,L,M,Mo,O |  | 335 | 1675 |  |
| *Asplenium lobulatum* | Ind | Fern | M,W | H,K,L,M,Mo,O |  | 240 | 1525 |  |
| *Asplenium normale* | Ind | Fern | M,W | H,K,L,M,Mo,O |  | 375 | 1680 |  |
| *Asplenium polyodon* | Ind | Fern | M,W | H,K,L,M,Mo,O |  | 600 | 2000 |  |
| *Asplenium trichomanes* | End | Fern | D | H,M |  | 1200 | 2700 |  |
| *Astelia menziesiana* | End | Herb | M,W | H,K,L,M,Mo,O | Bird | 610 | 2230 | 26.7 |
| *Athyrium microphyllum* | End | Fern | M,W | H,K,L,M,Mo,O |  | 500 | 2320 |  |
| *Axonopus fissifolius* | Non | Grass | W | H,K,L,M,Mo,O | Other | 160 | 1220 | 6.3 |
| *Broussaisia arguta* | End | Shrub | M,W | H,K,L,M,Mo,O | Bird | 400 | 1400 | 10.1 |
| *Bulbostylis capillaris* | Non | Grass | D | H | Other | 100 | 1780 | 10.0 |
| *Carex alligata* | End | Grass | W | H,K,L,M,Mo,O | Other | 730 | 2225 | 4.7 |
| *Carex wahuensis* | End | Grass | D,M | H,K,L,M,Mo,O | Other | 250 | 2500 | 4.0 |
| *Cenchrus clandestinus* | Non | Grass | D,M | H,K,L,M,Mo,O | Other | 0 | 1220 | 7.5 |
| *Centaurium erythraea* | Non | Herb | D,M | H,K,L,M,Mo,O | Other | 10 | 2140 | 12.5 |
| *Chamaecrista nictitans* | Non | Herb | D,M | H,K,L,M,Mo,O | Other | 3 | 1130 | 6.0 |
| *Cheirodendron trigynum* | End | Tree | M,W | H,K,L,M,Mo,O | Bird | 310 | 2190 | 12.0 |
| *Cibotium glaucum* | End | Tree | M,W | H,K,L,M,Mo,O |  | 300 | 1700 |  |
| *Cibotium menziesii* | End | Tree | M,W | H,K,L,M,Mo,O |  | 250 | 1400 |  |
| *Cirsium vulgare* | Non | Herb | D,M | H,K,L,M,Mo,O | Wind |  |  | 5.3 |
| *Clermontia arborescens* | End | Tree | M,W | L,M,Mo | Bird | 520 | 1825 | 7.5 |
| *Clidemia hirta* | Non | Shrub | M,W | H,K,L,M,Mo,O | Bird |  |  | 8.5 |
| *Conyza bonariensis* | Non | Herb | D,M,W | H,K,L,M,Mo,O | Wind |  |  | 4.0 |
| *Coprosma ernodeoides* | End | Shrub | D | H,M | Bird | 1220 | 2590 | 4.3 |
| *Coprosma foliosa* | End | Shrub | M,W | K,L,M,Mo,O | Bird | 300 | 1830 | 33.0 |
| *Coprosma montana* | End | Shrub | D | H,M,Mo | Bird | 1830 | 3050 | 10.0 |
| *Coprosma ochracea* | End | Tree | W | H,L,M,Mo,O | Bird | 720 | 2290 | 29.2 |
| *Crepis capillaris* | Non | Herb | D,M | H | Wind | 1140 | 2260 | 90.0 |
| *Cuphea carthagenensis* | Non | Herb | M,W | H,K,L,M,Mo,O | Other | 50 | 935 | 6.5 |
| *Cyclosorus dentatus* | Non | Fern | M,W | H,K,L,M,Mo,O |  | 10 | 1250 |  |
| *Cyclosorus sandwicensis* | End | Fern | M,W | H,K,L,M,Mo,O |  | 750 | 2100 |  |
| *Cyperus polystachyos* | Ind | Grass | M,W | H,K,L,M,Mo,O | Other | 0 | 1420 | 5.0 |
| *Cyperus sanguinolentus* | Non | Grass | W | H,O | Other | 900 | 1000 | 48.0 |
| *Dactylis glomerata* | Non | Grass | M | H,K,L,M,Mo,O | Other | 100 | 2290 | 31.5 |
| *Deparia petersenii* | Non | Fern | M,W | H,K,L,M,Mo,O |  | 170 | 1325 |  |
| *Deschampsia nubigena* | End | Grass | M,W | H,K,M,Mo | Other | 600 | 2830 | 18.0 |
| *Desmodium incanum* | Non | Shrub | D,M | H,L,M,Mo,O | Bird | 5 | 460 | 6.8 |
| *Desmodium triflorum* | Non | Herb | D | H,K,L,M,Mo,O | Other | 2 | 440 | 14.7 |
| *Dianella sandwicensis* | Ind | Grass | D,M,W | H,K,L,M,Mo,O | Bird | 120 | 2140 | 5.0 |
| *Dianthus armeria* | Non | Herb | D,M | H | Wind | 1200 | 1450 | 5.0 |
| *Dicranopteris linearis* | Ind | Fern | M,W | H,K,L,M,Mo,O |  | 0 | 2000 |  |
| *Diplazium sandwichianum* | End | Fern | M,W | H,K,L,M,Mo,O |  | 105 | 1850 |  |
| *Diplopterygium pinnatum* | End | Fern | W | H,K,L,M,Mo,O |  | 350 | 1500 |  |
| *Dodonaea viscosa* | Ind | Shrub | D,M,W | H,K,L,M,Mo,O | Wind | 3 | 2350 | 10.0 |
| *Dryopteris glabra* | End | Fern | M,W | H,K,L,M,Mo,O |  | 460 | 2200 |  |
| *Dryopteris rubiginosa* | End | Fern | M,W | H,K,L,M,Mo,O |  | 885 | 2180 |  |
| *Dryopteris wallichiana* | Ind | Fern | mesic | H,K,M,Mo,O |  | 850 | 2740 |  |
| *Dubautia ciliolata* | End | Shrub | D | H | Wind | 900 | 3200 | 48.8 |
| *Dubautia menziesii* | End | Shrub | D | M | Wind | 1800 | 3075 | 14.4 |
| *Ehrharta stipoides* | Non | Grass | W | H,K,M,Mo,O | Other | 20 | 1400 | 4.0 |
| *Elaphoglossum crassifolium* | End | Fern | M,W | H,K,L,M,Mo,O |  | 300 | 1300 |  |
| *Elaphoglossum paleaceum* | Ind | Fern | M,W | H,K,M,Mo,O |  | 875 | 2075 |  |
| *Elaphoglossum parvisquameum* | End | Fern | W | H,L,M,Mo |  | 450 | 1800 |  |
| *Elaphoglossum wawrae* | End | Fern | M,W | H,K,M,Mo,O |  | 800 | 2100 |  |
| *Emilia fosbergii* | Non | Herb | D | H,K,L,M,Mo,O | Wind | 0 | 1000 | 26.3 |
| *Eragrostis brownei* | Non | Grass | M,W | H,K,M,Mo | Other | 1130 | 2000 | 7.5 |
| *Euchiton sphaericus* | Non | Herb | D,M,W | H,L,M,Mo | Wind | 600 | 2800 | 49.0 |
| *Festuca bromoides* | Non | Grass | D | H,K,L,M,Mo,O | Other | 185 | 2590 |  |
| *Festuca rubra* | Non | Grass | D,M | H,K,M,Mo | Other | 820 | 2140 |  |
| *Fragaria vesca* | Non | Herb | M,W | H,K | Bird | 730 | 1700 | 20.0 |
| *Freycinetia arborea* | Ind | Shrub | M,W | H,K,L,M,Mo,O | Bird | 300 | 1500 | 6.0 |
| *Geranium cuneatum* | End | Shrub | D,M | H,M | Other | 1480 | 3250 | 3.4 |
| *Geranium homeanum* | Non | Herb | M | H,K,M | Other | 1130 | 2150 | 6.0 |
| *Grammitis hookeri* | Ind | Fern | M,W | H,K,L,M,Mo,O |  | 800 | 1750 |  |
| *Hedychium gardnerianum* | Non | Herb | W | H,K,L,M,O | Bird |  |  | 3.4 |
| *Heteropogon contortus* | Ind | Grass | D | H,K,L,M,Mo,O | Other | 0 | 700 | 7.0 |
| *Holcus lanatus* | Non | Grass | W | H,K,L,M,Mo,O | Other | 760 | 3250 | 4.0 |
| *Hymenophyllum lanceolatum* | End | Fern | W | H,K,L,M,Mo,O |  | 440 | 1320 |  |
| *Hymenophyllum recurvum* | End | Fern | W | H,K,L,M,Mo,O |  | 270 | 1525 |  |
| *Hyparrhenia rufa* | Non | Grass | D | H,K,M,Mo,O | Other | 10 | 660 | 8.0 |
| *Hypochoeris radicata* | Non | Herb | D,M,W | H,K,L,M,Mo,O | Wind | 1100 | 2800 | 163.3 |
| *Ilex anomala* | Ind | Tree | M,W | H,K,L,M,Mo,O | Bird | 600 | 1400 | 9.0 |
| *Indigofera suffruticosa* | Non | Shrub | D | H,K,L,M,Mo,O | Other | 3 | 1160 | 19.0 |
| *Isachne distichophylla* | End | Grass | W | H,K,L,M,Mo,O | Other | 215 | 1480 | 10.2 |
| *Juncus effusus* | Non | Grass | W | H,M,Mo | Other | 1000 | 2000 |  |
| *Kadua affinis* | End | Tree | M,W | H,K,L,M,Mo,O | Bird | 260 | 2040 | 90.0 |
| *Kadua axillaris* | End | Shrub | M,W | H,M,Mo | Bird | 400 | 1830 | 6.3 |
| *Kyllinga brevifolia* | Non | Grass | M,W | H,K,L,M,Mo,O | Other | 20 | 1220 | 6.4 |
| *Labordia hedyosmifolia* | End | Shrub | M,W | H,L,M,Mo | Bird | 700 | 1590 | 8.3 |
| *Lantana camara* | Non | Shrub | D,M | H,K,L,M,Mo,O | Bird | 2 | 1070 | 13.5 |
| *Lepisorus thunbergianus* | Ind | Fern | D,M,W | H,K,L,M,Mo,O |  | 10 | 2100 |  |
| *Leptecophylla tameiameiae* | Ind | Shrub | D,M,W | H,K,L,M,Mo,O | Bird | 15 | 3230 | 8.3 |
| *Liparis hawaiensis* | End | Herb | W | H,K,L,M,Mo,O | Wind | 1490 | 1530 | 6.3 |
| *Lotus uliginosus* | Non | Herb | W | H,K,M,Mo | Other |  |  | 15.6 |
| *Luzula hawaiiensis* | End | Grass | D | H,K,L,M,Mo,O | Other | 730 | 2560 | 60.0 |
| *Lycopodiella cernua* | Ind | Fern | M,W | H,K,L,M,Mo,O |  | 15 | 2135 |  |
| *Lycopodium venustulum* | Ind | Fern | M,W | H,K,L,M,Mo,O |  | 825 | 2135 |  |
| *Lythrum maritimum* | Non | Shrub | M,W | H,K,L,M,Mo,O | Other | 0 | 2450 | 33.0 |
| *Machaerina angustifolia* | Ind | Grass | W | H,K,L,M,Mo,O | Other | 420 | 2070 | 5.2 |
| *Medicago lupulina* | Non | Herb | D,M | H,M,O | Other | 5 | 2840 | 32.0 |
| *Melicope clusiifolia* | End | Tree | M,W | H,K,L,M,Mo,O | Bird |  |  | 14.4 |
| *Melinis minutiflora* | Non | Grass | D,M | H,K,L,M,Mo,O | Other | 120 | 1220 | 9.2 |
| *Melinis repens* | Non | Grass | D | H,K,L,M,Mo,O | Other | 0 | 1950 | 30.0 |
| *Metrosideros polymorpha* | End | Tree | D,M,W | H,K,L,M,Mo,O | Wind | 0 | 2200 | 44.0 |
| *Microlepia strigosa* | Ind | Fern | D,M,W | H,K,L,M,Mo,O |  | 0 | 1770 |  |
| *Morella faya* | Non | Tree | M,W | H,K,L,M,O | Bird | 150 | 1310 | 6.9 |
| *Morelotia gahniiformis* | End | Grass | D | H,L,M,Mo | Other | 520 | 2380 | 7.0 |
| *Myoporum sandwicense* | Ind | Shrub | D,M,W | H,K,L,M,Mo,O | Bird | 0 | 2380 | 50.3 |
| *Myrsine lessertiana* | End | Tree | D,M,W | H,K,L,M,Mo,O | Bird | 215 | 2200 | 9.2 |
| *Myrsine sandwicensis* | End | Tree | M,W | H,K,L,M,Mo,O | Bird | 300 | 1470 | 10.0 |
| *Nephrolepis brownii* | Non | Fern | D,M | H,K,L,M,Mo,O |  |  |  |  |
| *Nephrolepis cordifolia* | Ind | Fern | M,W | H,K,L,M,Mo,O |  | 440 | 1525 |  |
| *Nertera granadensis* | Ind | Herb | W | H,K,M,Mo,O | Bird | 490 | 2070 | 24.0 |
| *Oenothera stricta* | Non | Herb | D | H,M | Other | 1200 | 2740 | 2.4 |
| *Osteomeles anthyllidifolia* | Ind | Shrub | D,M | H,K,L,M,Mo,O | Bird | 2 | 2320 | 3.5 |
| *Oxalis corniculata* | Non | Herb | D | H,K,L,M,Mo,O | Other | 0 | 2300 | 20.0 |
| *Paspalum conjugatum* | Non | Grass | M,W | H,K,L,M,Mo,O | Other | 10 | 950 | 7.0 |
| *Paspalum dilatatum* | Non | Grass | D,M,W | H,K,L,M,Mo,O | Other | 40 | 1620 | 6.0 |
| *Pellaea ternifolia* | Ind | Fern | D | H,K,L,M,Mo,O |  | 600 | 3500 |  |
| *Peperomia cookiana* | End | Herb | M,W | H,K,M,Mo | Other | 460 | 1980 | 25.0 |
| *Perrottetia sandwicensis* | End | Tree | W | H,K,L,M,Mo,O | Bird | 300 | 1250 | 7.2 |
| *Phymatosorus grossus* | Non | Fern | D,M,W | H,K,L,M,Mo,O |  | 0 | 600 |  |
| *Pipturus albidus* | End | Shrub | M,W | H,K,L,M,Mo,O | Bird | 70 | 1870 | 14.3 |
| *Plantago lanceolata* | Non | Herb | D | H,K,L,M,Mo,O | Bird | 0 | 3110 | 64.0 |
| *Pluchea carolinensis* | Non | Shrub | D,M,W | H,K,L,M,Mo,O | Wind | 0 | 900 | 16.0 |
| *Polypodium pellucidum* | End | Fern | M,W | H,K,L,M,Mo,O |  | 150 | 2135 |  |
| *Prunella vulgaris* | Non | Herb | M,W | H,M,Mo | Other | 820 | 2260 | 25.7 |
| *Pseudognaphalium sandwicensium* | End | Herb | D | H,K,L,M,Mo,O | Wind | 0 | 3000 | 130.0 |
| *Psidium cattleianum* | Non | Tree | M,W | H,K,L,M,Mo,O | Bird | 15 | 1220 | 7.7 |
| *Psidium guajava* | Non | Shrub | D,M,W | H,K,L,M,Mo,O | Bird | 15 | 1220 | 4.4 |
| *Psilotum complanatum* | Ind | Fern | M,W | H,K,L,M,Mo,O |  | 250 | 1100 |  |
| *Psilotum nudum* | Ind | Fern | M,W | H,K,L,M,Mo,O |  | 0 | 1200 |  |
| *Psychotria hawaiiensis* | End | Tree | M,W | H,M,Mo | Bird | 150 | 1590 | 36.9 |
| *Pteridium aquilinum* | End | Fern | M,W | H,K,L,M,Mo,O |  | 300 | 2700 |  |
| *Pteris cretica* | Ind | Fern | D,M | H,K,L,M,Mo,O |  | 340 | 2450 |  |
| *Rubus argutus* | Non | Shrub | M,W | H,K,L,M,Mo,O | Bird | 200 | 2300 | 2.3 |
| *Rubus ellipticus* | Non | Shrub | W | H,Mo | Bird | 1060 | 1220 | 1.7 |
| *Rubus hawaiensis* | End | Shrub | D,M,W | H,K,M,Mo | Bird | 660 | 3070 | 3.8 |
| *Rubus rosifolius* | Non | Shrub | M,W | H,K,L,M,Mo,O | Bird | 60 | 1730 | 16.0 |
| *Rumex acetosella* | Non | Herb | D,M | H,K,M | Wind | 1115 | 2840 | 4.0 |
| *Sacciolepis indica* | Non | Grass | W | H,K,M,Mo,O | Other | 150 | 1700 | 6.7 |
| *Sadleria cyatheoides* | End | Tree | D,M,W | H,K,L,M,Mo,O |  | 75 | 2200 |  |
| *Sadleria pallida* | End | Tree | W | H,K,L,M,Mo,O |  | 25 | 2150 |  |
| *Schinus terebinthifolius* | Non | Tree | D,M | H,K,L,M,Mo,O | Bird | 3 | 920 | 22.9 |
| *Schizachyrium condensatum* | Non | Grass | M | H,K,M,Mo,O | Other | 210 | 1310 | 10.7 |
| *Senecio madagascariensis* | Non | Herb | M,W | H,K,L,M,Mo,O | Wind |  |  |  |
| *Setaria palmifolia* | Non | Grass | M,W | H,K,L,M,Mo,O | Other | 240 | 1160 | 45.0 |
| *Setaria parviflora* | Non | Grass | D,M,W | H,K,L,M,Mo,O | Other | 0 | 1480 | 12.5 |
| *Smilax melastomifolia* | End | Shrub | M,W | H,K,L,M,Mo,O | Bird | 180 | 2080 | 11.9 |
| *Sophora chrysophylla* | End | Shrub | D,M,W | H,K,L,M,Mo,O | Bird | 450 | 3240 | 54.8 |
| *Sphenomeris chinensis* | Ind | Fern | M,W | H,K,L,M,Mo,O |  | 40 | 1310 |  |
| *Sporobolus africanus* | Non | Grass | M | H,K,L,M,Mo,O | Other | 150 | 2440 | 6.3 |
| *Syzygium cumini* | Non | Tree | M | H,K,L,M,Mo,O | Bird | 40 | 1230 | 11.9 |
| *Tetramolopium humile* | End | Shrub | D | H,M | Wind | 1900 | 3300 | 9.4 |
| *Thelypteris globulifera* | End | Fern | W | H,K,L,M,Mo,O |  | 100 | 2200 |  |
| *Tibouchina herbacea* | Non | Herb | M,W | H,M | Bird |  |  | 6.7 |
| *Trisetum glomeratum* | End | Grass | D | H,M | Other | 750 | 4090 | 5.0 |
| *Uncinia uncinata* | Ind | Grass | W | H,K,M,Mo | Bird | 1040 | 2100 | 4.3 |
| *Vaccinium calycinum* | End | Shrub | W | H,K,L,M,Mo,O | Bird | 500 | 1800 | 3.2 |
| *Vaccinium reticulatum* | End | Shrub | D | H,K,L,M,Mo,O | Bird | 640 | 3700 | 9.0 |
| *Vandenboschia davallioides* | End | Fern | M,W | H,K,L,M,Mo,O |  | 250 | 1800 |  |
| *Veronica serpyllifolia* | Non | Herb | D,M,W | H,K,L,M,Mo | Wind | 820 | 3060 | 7.0 |
| *Waltheria indica* | Ind | Shrub | D | H,K,L,M,Mo,O | Other | 0 | 1220 | 46.5 |
| *Wikstroemia oahuensis* | End | Shrub | M,W | K,L,M,Mo,O | Bird | 100 | 1400 | 6.4 |
| *Wikstroemia phillyreifolia* | End | Shrub | D,M | H | Bird | 0 | 2290 | 3.0 |

**References**

Wagner WL, Herbst DR, Sohmer SH. Manual of the flowering plants of Hawaii. 2^nd^ ed. Honolulu: University of Hawaii Press and Bishop Museum Press; 1999.

Palmer DD. Hawaii's ferns and fern allies. Honolulu: University of Hawaii Press; 2003.

Wagner WL, Herbst DR, Kahn N, Flynn T. Hawaiian vascular plant updates: a supplement to the manual of flowering plants of Hawaii and Hawaii's ferns and fern allies. Honolulu: University of Hawaii Press; 2012.
