# Supplementary material for: Classifying Hawaiian plant species along a habitat generalist-specialist continuum: Implications for species conservation under climate change
Source: PLoS One. 2020 Feb 7;15(2):e0228573. doi: 10.1371/journal.pone.0228573 (PMC7006925; doi:10.1371/journal.pone.0228573)
Supplement: S2 Appendix — (DOCX) [file pone.0228573.s002.docx]

**S2 Appendix. Habitat specialization rankings for the 170 plant species that met the 25-plot occurrence cut-off.**

Species Jaccard index values from most generalist to most specialist rankings. For each species, the Jaccard index value (standard deviation (SD) based on 100 permutations of selecting 25 plots), mean alpha richness per plot listed as μ(α), total number of plot occurrences out of 1019 possible, species origin, and life form are provided.

| **Species** | **Jaccard index ± SD** | | | **μ(α)** | **Occurrence** | **Origin** | **Life form** |
| --- | --- | --- | --- | --- | --- | --- | --- |
| *Oxalis corniculata* | 0.895 | ± | 0.007 | 15.8 | 53 | Non-Native | Herb |
| *Ageratina riparia* | 0.888 | ± | 0.008 | 20.3 | 72 | Non-Native | Shrub |
| *Psidium cattleianum* | 0.884 | ± | 0.016 | 21.7 | 137 | Non-Native | Tree |
| *Conyza bonariensis* | 0.882 | ± | 0.007 | 18.6 | 29 | Non-Native | Herb |
| *Ageratum conyzoides* | 0.879 | ± | 0.009 | 13.3 | 31 | Non-Native | Herb |
| *Metrosideros polymorpha* | 0.876 | ± | 0.013 | 19.6 | 586 | Endemic | Tree |
| *Schinus terebinthifolius* | 0.875 | ± | 0.009 | 14.2 | 52 | Non-Native | Tree |
| *Lantana camara* | 0.874 | ± | 0.014 | 12.1 | 75 | Non-Native | Shrub |
| *Ageratina adenophora* | 0.873 | ± | 0.011 | 24.8 | 50 | Non-Native | Shrub |
| *Psidium guajava* | 0.871 | ± | 0.012 | 13.8 | 64 | Non-Native | Shrub |
| *Cyclosorus dentatus* | 0.869 | ± | 0.010 | 18.2 | 38 | Non-Native | Fern |
| *Psilotum nudum* | 0.866 | ± | 0.020 | 24.8 | 66 | Indigenous | Fern |
| *Nephrolepis brownii* | 0.863 | ± | 0.017 | 15.0 | 135 | Non-Native | Fern |
| *Ehrharta stipoides* | 0.860 | ± | 0.012 | 20.4 | 135 | Non-Native | Grass |
| *Emilia fosbergii* | 0.857 | ± | 0.010 | 14.8 | 25 | Non-Native | Herb |
| *Rubus rosifolius* | 0.854 | ± | 0.020 | 27.9 | 92 | Non-Native | Shrub |
| *Acacia koa* | 0.854 | ± | 0.020 | 22.2 | 110 | Endemic | Tree |
| *Sphenomeris chinensis* | 0.853 | ± | 0.013 | 27.5 | 44 | Indigenous | Fern |
| *Cenchrus clandestinus* | 0.853 | ± | 0.020 | 16.7 | 140 | Non-Native | Grass |
| *Rubus argutus* | 0.852 | ± | 0.011 | 19.7 | 45 | Non-Native | Shrub |
| *Syzygium cumini* | 0.845 | ± | 0.008 | 11.4 | 44 | Non-Native | Tree |
| *Sadleria cyatheoides* | 0.843 | ± | 0.013 | 19.7 | 49 | Endemic | Tree |
| *Leptecophylla tameiameiae* | 0.842 | ± | 0.021 | 16.2 | 517 | Indigenous | Shrub |
| *Axonopus fissifolius* | 0.839 | ± | 0.022 | 22.4 | 83 | Non-Native | Grass |
| *Desmodium incanum* | 0.839 | ± | 0.008 | 15.6 | 26 | Non-Native | Shrub |
| *Phymatosorus grossus* | 0.836 | ± | 0.009 | 13.8 | 30 | Non-Native | Fern |
| *Pseudognaphalium sandwicensium* | 0.829 | ± | 0.015 | 16.6 | 40 | Endemic | Herb |
| *Myoporum sandwicense* | 0.828 | ± | 0.010 | 15.9 | 30 | Indigenous | Shrub |
| *Pluchea carolinensis* | 0.827 | ± | 0.023 | 13.3 | 39 | Non-Native | Shrub |
| *Sporobolus africanus* | 0.825 | ± | 0.021 | 16.7 | 130 | Non-Native | Grass |
| *Microlepia strigosa* | 0.825 | ± | 0.023 | 25.0 | 43 | Indigenous | Fern |
| *Cibotium glaucum* | 0.824 | ± | 0.019 | 27.1 | 260 | Endemic | Tree |
| *Setaria parviflora* | 0.824 | ± | 0.010 | 18.3 | 37 | Non-Native | Grass |
| *Cirsium vulgare* | 0.823 | ± | 0.015 | 19.7 | 46 | Non-Native | Herb |
| *Pipturus albidus* | 0.822 | ± | 0.011 | 23.3 | 43 | Endemic | Shrub |
| *Machaerina angustifolia* | 0.822 | ± | 0.014 | 20.1 | 73 | Indigenous | Grass |
| *Tibouchina herbacea* | 0.821 | ± | 0.013 | 24.7 | 45 | Non-Native | Herb |
| *Paspalum conjugatum* | 0.821 | ± | 0.020 | 25.6 | 76 | Non-Native | Grass |
| *Polypodium pellucidum* | 0.820 | ± | 0.014 | 27.1 | 125 | Endemic | Fern |
| *Sacciolepis indica* | 0.819 | ± | 0.026 | 21.5 | 48 | Non-Native | Grass |
| *Dicranopteris linearis* | 0.819 | ± | 0.018 | 27.4 | 145 | Indigenous | Fern |
| *Kyllinga brevifolia* | 0.818 | ± | 0.024 | 18.5 | 51 | Non-Native | Grass |
| *Clidemia hirta* | 0.817 | ± | 0.027 | 25.2 | 66 | Non-Native | Shrub |
| *Dryopteris wallichiana* | 0.814 | ± | 0.027 | 26.0 | 149 | Indigenous | Fern |
| *Wikstroemia oahuensis* | 0.812 | ± | 0.018 | 33.4 | 30 | Endemic | Shrub |
| *Carex wahuensis* | 0.811 | ± | 0.019 | 15.1 | 112 | Endemic | Grass |
| *Dodonaea viscosa* | 0.808 | ± | 0.021 | 13.3 | 309 | Indigenous | Shrub |
| *Sophora chrysophylla* | 0.807 | ± | 0.023 | 16.0 | 127 | Endemic | Shrub |
| *Lepisorus thunbergianus* | 0.803 | ± | 0.031 | 31.7 | 54 | Indigenous | Fern |
| *Vaccinium reticulatum* | 0.803 | ± | 0.025 | 16.0 | 299 | Endemic | Shrub |
| *Cyperus polystachyos* | 0.801 | ± | 0.023 | 17.1 | 67 | Indigenous | Grass |
| *Anthoxanthum odoratum* | 0.800 | ± | 0.029 | 17.3 | 188 | Non-Native | Grass |
| *Indigofera suffruticosa* | 0.799 | ± | 0.020 | 12.1 | 31 | Non-Native | Shrub |
| *Vaccinium calycinum* | 0.797 | ± | 0.020 | 28.8 | 205 | Endemic | Shrub |
| *Pteridium aquilinum* | 0.796 | ± | 0.025 | 14.1 | 166 | Endemic | Fern |
| *Holcus lanatus* | 0.792 | ± | 0.025 | 16.7 | 194 | Non-Native | Grass |
| *Hypochoeris radicata* | 0.792 | ± | 0.029 | 16.4 | 213 | Non-Native | Herb |
| *Rumex acetosella* | 0.790 | ± | 0.020 | 17.1 | 118 | Non-Native | Herb |
| *Anagallis arvensis* | 0.790 | ± | 0.031 | 17.9 | 36 | Non-Native | Herb |
| *Cheirodendron trigynum* | 0.787 | ± | 0.019 | 29.0 | 246 | Endemic | Tree |
| *Myrsine lessertiana* | 0.786 | ± | 0.023 | 28.9 | 178 | Endemic | Tree |
| *Juncus effusus* | 0.781 | ± | 0.022 | 30.1 | 45 | Non-Native | Grass |
| *Paspalum dilatatum* | 0.777 | ± | 0.014 | 17.9 | 25 | Non-Native | Grass |
| *Asplenium trichomanes* | 0.776 | ± | 0.017 | 18.1 | 68 | Endemic | Fern |
| *Elaphoglossum crassifolium* | 0.776 | ± | 0.010 | 27.5 | 28 | Endemic | Fern |
| *Veronica serpyllifolia* | 0.776 | ± | 0.026 | 19.7 | 64 | Non-Native | Herb |
| *Trisetum glomeratum* | 0.775 | ± | 0.018 | 14.8 | 101 | Endemic | Grass |
| *Dryopteris glabra* | 0.774 | ± | 0.023 | 33.5 | 93 | Endemic | Fern |
| *Prunella vulgaris* | 0.774 | ± | 0.010 | 19.3 | 26 | Non-Native | Herb |
| *Hedychium gardnerianum* | 0.774 | ± | 0.020 | 30.2 | 66 | Non-Native | Herb |
| *Andropogon virginicus* | 0.773 | ± | 0.032 | 13.5 | 164 | Non-Native | Grass |
| *Lycopodiella cernua* | 0.771 | ± | 0.020 | 29.9 | 40 | Indigenous | Fern |
| *Alyxia stellata* | 0.768 | ± | 0.019 | 32.9 | 80 | Endemic | Shrub |
| *Geranium homeanum* | 0.768 | ± | 0.025 | 21.1 | 51 | Non-Native | Herb |
| *Ilex anomala* | 0.768 | ± | 0.018 | 29.2 | 162 | Indigenous | Tree |
| *Chamaecrista nictitans* | 0.768 | ± | 0.035 | 11.7 | 79 | Non-Native | Herb |
| *Deparia petersenii* | 0.766 | ± | 0.020 | 36.4 | 39 | Non-Native | Fern |
| *Melinis minutiflora* | 0.766 | ± | 0.034 | 12.9 | 125 | Non-Native | Grass |
| *Rubus hawaiensis* | 0.765 | ± | 0.026 | 27.2 | 83 | Endemic | Shrub |
| *Desmodium triflorum* | 0.763 | ± | 0.041 | 12.5 | 35 | Non-Native | Herb |
| *Melicope clusiifolia* | 0.762 | ± | 0.021 | 31.5 | 95 | Endemic | Tree |
| *Broussaisia arguta* | 0.762 | ± | 0.017 | 32.4 | 175 | Endemic | Shrub |
| *Cibotium menziesii* | 0.762 | ± | 0.021 | 32.9 | 147 | Endemic | Tree |
| *Elaphoglossum paleaceum* | 0.761 | ± | 0.018 | 32.3 | 161 | Indigenous | Fern |
| *Lycopodium venustulum* | 0.760 | ± | 0.021 | 25.2 | 47 | Indigenous | Fern |
| *Diplazium sandwichianum* | 0.757 | ± | 0.019 | 33.5 | 109 | Endemic | Fern |
| *Osteomeles anthyllidifolia* | 0.757 | ± | 0.025 | 14.2 | 65 | Indigenous | Shrub |
| *Smilax melastomifolia* | 0.757 | ± | 0.020 | 27.7 | 45 | Endemic | Shrub |
| *Morella faya* | 0.756 | ± | 0.022 | 14.6 | 50 | Non-Native | Tree |
| *Deschampsia nubigena* | 0.755 | ± | 0.026 | 15.1 | 240 | Endemic | Grass |
| *Freycinetia arborea* | 0.755 | ± | 0.023 | 32.7 | 89 | Indigenous | Shrub |
| *Carex alligata* | 0.755 | ± | 0.024 | 31.5 | 82 | Endemic | Grass |
| *Psychotria hawaiiensis* | 0.755 | ± | 0.015 | 26.4 | 38 | Endemic | Tree |
| *Sadleria pallida* | 0.755 | ± | 0.024 | 32.4 | 143 | Endemic | Tree |
| *Isachne distichophylla* | 0.755 | ± | 0.010 | 18.5 | 31 | Endemic | Grass |
| *Agrostis sandwicensis* | 0.753 | ± | 0.012 | 17.1 | 34 | Endemic | Grass |
| *Elaphoglossum wawrae* | 0.753 | ± | 0.021 | 29.5 | 124 | Endemic | Fern |
| *Coprosma ochracea* | 0.752 | ± | 0.020 | 31.2 | 48 | Endemic | Tree |
| *Pteris cretica* | 0.752 | ± | 0.028 | 19.7 | 32 | Indigenous | Fern |
| *Athyrium microphyllum* | 0.751 | ± | 0.021 | 33.2 | 146 | Endemic | Fern |
| *Centaurium erythraea* | 0.750 | ± | 0.012 | 17.6 | 65 | Non-Native | Herb |
| *Kadua affinis* | 0.748 | ± | 0.021 | 34.8 | 99 | Endemic | Tree |
| *Coprosma foliosa* | 0.748 | ± | 0.025 | 32.4 | 55 | Endemic | Shrub |
| *Uncinia uncinata* | 0.746 | ± | 0.019 | 28.4 | 114 | Indigenous | Grass |
| *Euchiton sphaericus* | 0.746 | ± | 0.027 | 16.7 | 56 | Non-Native | Herb |
| *Myrsine sandwicensis* | 0.741 | ± | 0.018 | 32.5 | 47 | Endemic | Tree |
| *Eragrostis brownei* | 0.741 | ± | 0.030 | 16.2 | 58 | Non-Native | Grass |
| *Psilotum complanatum* | 0.741 | ± | 0.025 | 35.3 | 68 | Indigenous | Fern |
| *Asplenium adiantum-nigrum* | 0.740 | ± | 0.025 | 17.5 | 53 | Indigenous | Fern |
| *Dactylis glomerata* | 0.739 | ± | 0.025 | 16.7 | 91 | Non-Native | Grass |
| *Senecio madagascariensis* | 0.735 | ± | 0.028 | 17.4 | 86 | Non-Native | Herb |
| *Cuphea carthagenensis* | 0.733 | ± | 0.027 | 22.5 | 29 | Non-Native | Herb |
| *Asplenium polyodon* | 0.733 | ± | 0.021 | 34.0 | 57 | Indigenous | Fern |
| *Pellaea ternifolia* | 0.732 | ± | 0.021 | 14.0 | 111 | Indigenous | Fern |
| *Elaphoglossum parvisquameum* | 0.731 | ± | 0.016 | 32.1 | 27 | Endemic | Fern |
| *Plantago lanceolata* | 0.730 | ± | 0.030 | 18.3 | 79 | Non-Native | Herb |
| *Astelia menziesiana* | 0.728 | ± | 0.018 | 34.5 | 117 | Endemic | Herb |
| *Vandenboschia davallioides* | 0.726 | ± | 0.015 | 35.8 | 78 | Endemic | Fern |
| *Waltheria indica* | 0.726 | ± | 0.036 | 10.9 | 72 | Indigenous | Shrub |
| *Hymenophyllum lanceolatum* | 0.723 | ± | 0.016 | 34.5 | 67 | Endemic | Fern |
| *Adenophorus tamariscinus* | 0.723 | ± | 0.020 | 36.2 | 114 | Endemic | Fern |
| *Asplenium contiguum* | 0.722 | ± | 0.025 | 35.0 | 54 | Endemic | Fern |
| *Adenophorus tenellus* | 0.719 | ± | 0.014 | 34.8 | 69 | Endemic | Fern |
| *Fragaria vesca* | 0.719 | ± | 0.017 | 18.1 | 27 | Non-Native | Herb |
| *Schizachyrium condensatum* | 0.717 | ± | 0.032 | 12.4 | 124 | Non-Native | Grass |
| *Luzula hawaiiensis* | 0.716 | ± | 0.031 | 16.8 | 149 | Endemic | Grass |
| *Morelotia gahniiformis* | 0.715 | ± | 0.022 | 13.8 | 198 | Endemic | Grass |
| *Perrottetia sandwicensis* | 0.715 | ± | 0.021 | 33.1 | 64 | Endemic | Tree |
| *Festuca rubra* | 0.714 | ± | 0.025 | 15.5 | 37 | Non-Native | Grass |
| *Melinis repens* | 0.713 | ± | 0.032 | 10.9 | 109 | Non-Native | Grass |
| *Coprosma ernodeoides* | 0.712 | ± | 0.028 | 15.0 | 178 | Endemic | Shrub |
| *Nephrolepis cordifolia* | 0.708 | ± | 0.023 | 34.5 | 46 | Indigenous | Fern |
| *Hymenophyllum recurvum* | 0.707 | ± | 0.016 | 37.4 | 104 | Endemic | Fern |
| *Peperomia cookiana* | 0.702 | ± | 0.018 | 39.5 | 31 | Endemic | Herb |
| *Cyclosorus sandwicensis* | 0.701 | ± | 0.019 | 35.2 | 66 | Endemic | Fern |
| *Kadua axillaris* | 0.697 | ± | 0.012 | 37.4 | 34 | Endemic | Shrub |
| *Grammitis hookeri* | 0.695 | ± | 0.012 | 34.9 | 53 | Indigenous | Fern |
| *Lythrum maritimum* | 0.693 | ± | 0.024 | 15.4 | 35 | Non-Native | Shrub |
| *Adenophorus pinnatifidus* | 0.692 | ± | 0.019 | 36.2 | 82 | Endemic | Fern |
| *Asplenium lobulatum* | 0.688 | ± | 0.012 | 39.6 | 51 | Indigenous | Fern |
| *Adenophorus hymenophylloides* | 0.687 | ± | 0.019 | 40.4 | 30 | Endemic | Fern |
| *Coprosma montana* | 0.684 | ± | 0.030 | 16.7 | 82 | Endemic | Shrub |
| *Dianella sandwicensis* | 0.683 | ± | 0.020 | 14.3 | 29 | Indigenous | Grass |
| *Bulbostylis capillaris* | 0.683 | ± | 0.026 | 10.9 | 105 | Non-Native | Grass |
| *Clermontia arborescens* | 0.682 | ± | 0.024 | 36.0 | 51 | Endemic | Tree |
| *Dryopteris rubiginosa* | 0.681 | ± | 0.016 | 41.5 | 34 | Endemic | Fern |
| *Cyperus sanguinolentus* | 0.679 | ± | 0.018 | 18.5 | 34 | Non-Native | Grass |
| *Adenophorus tripinnatifidus* | 0.678 | ± | 0.010 | 39.7 | 32 | Endemic | Fern |
| *Tetramolopium humile* | 0.676 | ± | 0.014 | 14.0 | 26 | Endemic | Shrub |
| *Lotus uliginosus* | 0.671 | ± | 0.020 | 18.8 | 31 | Non-Native | Herb |
| *Oenothera stricta* | 0.671 | ± | 0.017 | 16.3 | 26 | Non-Native | Herb |
| *Wikstroemia phillyreifolia* | 0.671 | ± | 0.022 | 12.9 | 34 | Endemic | Shrub |
| *Festuca bromoides* | 0.663 | ± | 0.023 | 18.7 | 59 | Non-Native | Grass |
| *Diplopterygium pinnatum* | 0.659 | ± | 0.012 | 39.9 | 32 | Endemic | Fern |
| *Rubus ellipticus* | 0.651 | ± | 0.017 | 34.2 | 29 | Non-Native | Shrub |
| *Nertera granadensis* | 0.650 | ± | 0.017 | 39.5 | 57 | Indigenous | Herb |
| *Setaria palmifolia* | 0.636 | ± | 0.010 | 34.2 | 28 | Non-Native | Grass |
| *Asplenium normale* | 0.633 | ± | 0.016 | 37.8 | 32 | Indigenous | Fern |
| *Labordia hedyosmifolia* | 0.627 | ± | 0.015 | 41.8 | 36 | Endemic | Shrub |
| *Dubautia ciliolata* | 0.620 | ± | 0.010 | 13.0 | 30 | Endemic | Shrub |
| *Medicago lupulina* | 0.615 | ± | 0.010 | 13.8 | 29 | Non-Native | Herb |
| *Thelypteris globulifera* | 0.610 | ± | 0.027 | 24.5 | 36 | Endemic | Fern |
| *Heteropogon contortus* | 0.593 | ± | 0.014 | 11.1 | 33 | Indigenous | Grass |
| *Dianthus armeria* | 0.592 | ± | 0.018 | 19.4 | 32 | Non-Native | Herb |
| *Hyparrhenia rufa* | 0.591 | ± | 0.020 | 9.8 | 41 | Non-Native | Grass |
| *Dubautia menziesii* | 0.587 | ± | 0.020 | 15.5 | 31 | Endemic | Shrub |
| *Liparis hawaiensis* | 0.582 | ± | 0.015 | 36.9 | 26 | Endemic | Herb |
| *Arrhenatherum elatius* | 0.578 | ± | 0.011 | 19.3 | 32 | Non-Native | Grass |
| *Geranium cuneatum* | 0.572 | ± | 0.023 | 16.2 | 40 | Endemic | Shrub |
| *Crepis capillaris* | 0.560 | ± | 0.014 | 19.8 | 27 | Non-Native | Herb |
