# Supplementary material for: Classifying Hawaiian plant species along a habitat generalist-specialist continuum: Implications for species conservation under climate change
Source: PLoS One. 2020 Feb 7;15(2):e0228573. doi: 10.1371/journal.pone.0228573 (PMC7006925; doi:10.1371/journal.pone.0228573)
Supplement: S3 Appendix — (DOCX) [file pone.0228573.s003.docx]

# **S3 Appendix. Relationship between habitat specialization rankings and species richness and species occurrence within plot data.**

## **Species richness**

Fridley and colleagues’ original methodology (2007) which was built on additive partitioning among plots, has been reviewed and criticized (Zelený 2009, Botta‐Dukát 2012) and subsequently revised (Manthey and Fridley 2009) to incorporate various forms of multiplicative partitioning to eliminate bias created by differences in species pool size across habitats. Removing this bias was particularly critical for Hawaiian plants, because some of the most common species (e.g., *Metrosideros polymorpha, Dodonaea viscosa, Leptecophylla tameiameiae*) are found in multiple habitats, with wide-ranging species pool sizes, across steep climatic/habitat gradients (i.e., <10 species in subalpine versus >40 species in wet forest plots). Mathematically, without this modification, only species found within the wet forest, the most species-rich type, would qualify as generalists, simply because these species occur in the most species rich plots. Our data still suggest some spurious autocorrelation among plant species restricted to wet forest environments with average alpha richness exceeding 26 species per plot (Fig. S3.A). Above this threshold, the community may be ecologically saturated or appear saturated due to sampling scheme, thereby limiting the effectiveness of co-occurrence data as a descriptor of habitat affinity for these species (Manthey and Fridley 2009).

**Figure S3.A.** For most species (61%), species pool size, measured as mean plot species richness μ(α), values are less than 26 species and no significant correlation was detected between the Jaccard index and richness (r = 0.23). However, for species found above the 26 species richness threshold, a strong negative correlation existed between Jaccard index values and increasing average plot richness (r = -0.78).

## **Species occurrence**

Differences in species occurrence within plot data were controlled for by randomly sampling and resampling 25 plots at a time. Twenty-five plots represents a balance between the quantity of plants analyzed and confidence in the resulting index (Fridley et al. 2007), regardless of how many occurrences each species had within the full data set. Species excluded from analysis owing to limited plot replication cannot be assumed to be habitat specialists since rarity may also be attributed to limited geographic range and/or local population size independent of habitat specificity (Rabinowitz 1981). The weak positive relationship we found between species habitat niche breadth and occurrence frequency (Fig. S3.B) does not necessarily imply erroneous results arising from methodological bias. The species with the highest occurrence frequencies, *M. polymorpha, L. tameiameiae, D. viscosa,* and *Vaccinium reticulatum*, have relatively high generalist scores, as expected given these species’ known existence across a broad spectrum of habitat types in Hawaiʻi (Wagner et al. 1999). Further, all four of these species (two endemic and two indigenous) have historically been divided and recombined as separate species and/or varieties owing to high morphological variability. *M. polymorpha*, the dominant tree in the islands, has long been divided into intraspecific varieties based on morphology (Stemmermann 1983, Mueller-Dombois and Fosberg 1998, Wagner et al. 1999). Initial genetic analyses validated three separate species on Oahʻu and suggested that *M. rugosa* and *M. tremuloides* represent recent and incipient speciation from *M. polymorpha* (Aradhya et al. 1991). Recent field and molecular analyses, have uncovered interesting thresholds, distribution boundaries, and parallel insular evolution for *Metrosideros* species (Harbaugh et al. 2009) and varieties (Stacy et al. 2014) which may help to explain this species’ remarkable ecological amplitude and subsequent wide habitat niche breadth. It is possible that similar molecular analyses for additional Hawaiian species will uncover evolutionary patterns that may divide other species into multiple taxa.

**Figure S3.B.** Species abundance, measured as the number of plot occurrences per species, was significantly (Pearson's product-moment correlation, p < 0.01) but weakly (r = 0.28) correlated with the Jaccard index (0.4 = specialist; 1 = complete generalist). Plot occurrence values are graphed on a log scale (base 2).

## **References**

Aradhya K, Mueller-Dombois D, Ranker T. Genetic evidence for recent and incipient speciation in the evolution of Hawaiian Metrosideros (Myrtaceae). Heredity. 1991;67:129-138.

Botta‐Dukát Z. Co‐occurrence‐based measure of species' habitat specialization: robust, unbiased estimation in saturated communities. Journal of Vegetation Science. 2012;23:201-207.

Fridley JD, Vandermast DB, Kuppinger DM, Manthey M, Peet RK. Co-occurrence based assessment of habitat generalists and specialists: a new approach for the measurement of niche width. Journal of Ecology. 2007;95:707-722.

Harbaugh DT, Wagner WL, Percy DM, James HF, Fleischer RC. Genetic Structure of the Polymorphic Metrosideros (Myrtaceae) Complex in the Hawaiian Islands Using Nuclear Microsatellite Data. PLoS ONE. 2009; 4:e4698. <https://doi.org/4610.1371/journal.pone.0004698>.

Manthey M, Fridley JD. Beta diversity metrics and the estimation of niche width via species co-occurrence data: reply to Zeleny. Journal of Ecology. 2009;97:18-22.

Mueller-Dombois D, Fosberg FR. Vegetation of the tropical Pacific islands. New York: Springer; 1998.

Rabinowitz D. Seven forms of rarity.in Synge H, editor. The biological aspects of rare plant conservation. New York: Wiley;1981.

Stacy E, Johansen J, Sakishima T, Price D, Pillon Y. Incipient radiation within the dominant Hawaiian tree Metrosideros polymorpha. Heredity. 2014;113:334-342.

Stemmermann L. Ecological studies of Hawaiian Metrosideros in a successional context. Pacific Science. 1983;37:361-373.

Wagner WL, Herbst DR, Sohmer SH. Manual of the flowering plants of Hawaii. 2^nd^ ed. Honolulu: University of Hawaii Press and Bishop Museum Press; 1999.

Zelený D. Co‐occurrence based assessment of species habitat specialization is affected by the size of species pool: reply to. Journal of Ecology. 2009;97:10-17.
